# Three Rice NAC Transcription Factors Heteromerize and Are Associated with Seed Size

**DOI:** 10.3389/fpls.2016.01638

**Published:** 2016-11-07

**Authors:** Iny Elizebeth Mathew, Sweta Das, Arunima Mahto, Pinky Agarwal

**Affiliations:** National Institute of Plant Genome ResearchNew Delhi, India

**Keywords:** association analysis, NAC, rice, seed development, transcriptional properties

## Abstract

NACs are plant-specific transcription factors (TFs) involved in multiple aspects of development and stress. In rice, three NAC TF encoding genes, namely *ONAC020, ONAC026*, and *ONAC023* express specifically during seed development, at extremely high levels. They exhibit significantly strong association with seed size/weight with the sequence variations located in the upstream regulatory region. Concomitantly, their expression pattern/levels during seed development vary amongst different accessions with variation in seed size. The alterations in the promoter sequences of the three genes, amongst the five rice accessions, correlate with the expression levels to a certain extent only. In terms of transcriptional properties, the three NAC TFs can activate and/or suppress downstream genes, though to different extents. Only ONAC026 is localized to the nucleus while ONAC020 and ONAC023 are targeted to the ER and cytoplasm, respectively. Interestingly, these two proteins interact with ONAC026 and the dimers localize in the nucleus. *Trans*-splicing between *ONAC020* and *ONAC026* results in three additional forms of *ONAC020*. The transcriptional properties including activation, repression, subcellular localization and heterodimerization of *trans-*spliced forms of ONAC020 and ONAC026 are different, indicating toward their role as competitors. The analysis presented in this paper helps to conclude that the three *NAC* genes, which are associated with seed size, have independent as well as overlapping roles during the process and can be exploited as potential targets for crop improvement.

## Introduction

Transcription factors (TFs) regulate the expression of the downstream target genes, in response to various external and/or internal stimuli, by binding to their upstream *cis* elements either as a monomer or a homodimer, or by interacting with other TFs or regulators. This specific binding is responsible for the spatial and temporal expression of the regulated genes, ultimately leading to a particular response. The functional specificity of these proteins is maintained by the presence of characteristic functional domains (Olsen et al., 2005; Agarwal et al., 2011). Rice seed development is regulated at the transcriptional level by a diverse group of TFs. Starch biosynthesis is controlled by OsBP-5, a MYC TF and OsEBP-89, an EREBP TF which act synergistically to regulate *Wx*, a starch synthase gene (Zhu et al., 2003). Another EREBP TF, rice starch regulator1 (RSR1), negatively regulates the expression of starch biosynthesis genes (Fu and Xue, 2010). A bZIP protein RISBZ1, and a DOF protein RPBF act synergistically in the regulation of storage protein synthesis and also affect starch biosynthesis in rice seeds (Kawakatsu et al., 2009). *OsNF-YB1*, an endosperm specific gene, is essential for proper endosperm development by regulating the cell proliferation genes (Sun et al., 2014). Similarly, *OsMADS6*, highly expressed in the flowers and endosperm, has been shown to be essential for normal endosperm development in rice (Zhang J. et al., 2010). OsMADS29 is a key regulator of early grain development in rice. The gene, expressing preferentially in the nucellus and nucellar projection, promotes programmed cell death of the maternal tissues (Yin and Xue, 2012; Nayar et al., 2013). OsWRKY78 has been shown to act as a seed development regulator in rice (Zhang et al., 2011). OsWRKY24, OsWRKY53, and OsWRKY70 act in a partially redundant manner in regulating GA and ABA signaling pathways in aleurone cells (Zhang L. et al., 2015). Grain width 8 (GW8)/OsSPL16 is a positive regulator of cell proliferation and controls grain width and yield in rice (Wang et al., 2012). Another QTL, *grain length and width2* (*GLW2*) encodes a growth regulating factor 4 (OsGRF4), which regulates grain weight and interacts with OsGIF1 (GRF interacting factor 1), another positive regulator of grain size in rice (Li et al., 2016).

NAC is one of the largest group of plant-specific TFs, named after the first three reported members of the family, NO APICAL MERISTEM (NAM), *Arabidopsis thaliana* Activation Factor1 and 2 (ATAF1/2) and CUP-SHAPED COTYLEDON 2 (CUC2) (Souer et al., 1996; Aida et al., 1997; Christianson et al., 2010). Each plant genome has multiple encoding members, with 117 and 151 genes in *Arabidopsis* and rice, respectively (Nuruzzaman et al., 2010), and similar numbers in other species as well (Pereira-Santana et al., 2015). They have a conserved NAC domain of about 150 amino acids followed by a diversified transcriptional regulatory region (TRR). The NAC domain is further categorized into five subdomains designated A–E, of which A, C and D are highly conserved. The N-terminal regions of these TFs hold a large number of charged amino acid residues. Subdomains C–E exhibit a net positive charge, while the remaining two are negatively charged. The high conservation of the C and D subdomains and the richness in basic amino acids suggests the involvement of these two regions in conferring the DNA binding property. TRR determines the activation or repression property of the protein and may also influence oligomerization property (Kikuchi et al., 2000; Ooka et al., 2003; Fang et al., 2008).

NAC TFs have been found to regulate a wide array of plant functions. During development, the redundant maternal proteins, *Arabidopsis* NARS1 and NARS2 control PCD of the inner integument of the ovule (Kunieda et al., 2008), and *SlNAC1, SlNAC4, SlNAC48*, and *SlNAC19* control tomato fruit ripening (Kou et al., 2016). *OsNAP* overexpression delays leaf senescence causing an increase in seed yield (Liang et al., 2014), while *OsNAC5* senses the senescence signal and is responsible for iron remobilization to the seeds (Ricachenevsky et al., 2013). In wheat, single nucleotide polymorphism (SNPs) and differential expression of *NAM-G1*, in 12 accessions, causes variation in grain protein content (Hu et al., 2013). Hence, NACs are an important class of TFs controlling various aspects of seed development (Agarwal et al., 2011) and their functions and sequences need to be explored further. A number of studies have successfully demonstrated the efficiency of a combinatorial strategy which combines high-resolution SNP-based and candidate gene-based association analysis, traditional genetic/QTL mapping, differential expression profiling and molecular haplotyping, for quantitative dissection of complex yield component traits in diverse crop plants, including rice (Zhao et al., 2011; Zuo and Li, 2014; Kujur et al., 2015a; Agarwal et al., 2016). It would be interesting to utilize this integrated approach to identify the functional alleles of *NAC* genes regulating grain size/weight variation, in cultivars adapted to diverse natural agro-climatic conditions. This can aid in genomics-assisted rice crop improvement.

In accordance with biological diversification, similar proteins are known to exhibit variation in properties as well as interact amongst themselves. Two closely related *NAC* TFs, with 66% identity in the protein sequences, control stomata differently. JA2 and JA2L cause stomatal closure and opening, respectively (Du et al., 2014) while *OsSWN1* and *2* control different aspects of secondary wall formation (Yoshida et al., 2013). Similarly, four phytoene synthase (*PSY*) genes of loquat function differently (Fu et al., 2014) though they cause carotenoid production. Penetration-resistance genes *PEN1, PEN2*, and *PEN3* function in overlapping as well as distinct manners in cell wall-based defense (Johansson et al., 2014). Closely related amino acid transporter genes, *AtCAT2, 3*, and *4* show overlap as well as distinctness in their subcellular localization as well as expression patterns (Yang et al., 2014). *SQUAMOSA promoter binding protein-like* genes, *GhSPL3* and *18* control flowering, second shoots and leaf development with *GhSOC1* binding to the promoter of *GhSPL3* but not *GhSPL18* (Zhang X. et al., 2015). Basic helix-loop-helix (bHLH) TF LONESOME HIGHWAY (LHW) interacts with two other members of the same TF family, *TARGET OF MONOPTEROS5* (*TMO5*) and *TMO5-LIKE1* (*T5L1*) to control various aspects of vasculature development (Ohashi-Ito et al., 2014). All the above examples stand testimony to the fact that genes from the same family, with overlapping expression and/or close phylogenetic relation, can control the same biological process both through independent and related pathways.

In order to deepen the knowledge about the role of NAC TFs in rice seed development, three seed-specific *NAC* genes have been chosen, on the basis of their expression pattern in diverse accessions and their phylogeny. Their promoter sequences have been compared in five accessions. Trait association mapping and association SNP analysis has been done for these genes to identify grain size/weight related allelic variants. Further, their transcriptional properties, including activation, repression, sub-cellular localization and heterodimerization have been examined. Transcriptional properties of multiple forms arising due to transcript fusion or *trans-*splicing among two of these genes have also been analyzed. In short, we have been able to assess the transcriptional properties of three NAC encoding genes, and their association with rice seed size/weight, and hence, put forth their method of regulation.

## Materials and Methods

### Cloning and Expression Profiling of Rice NAC Genes

Rice accessions with variable seed weights namely, *indica*/aromatics cv. Sonasal (SN), Pusa Basmati1 (PB1), *indica* Rice 64 (IR64) and Long Grain Rice (LGR), and a *japonica* cv. Nipponbare (NB) were grown in the field during crop growing season at NIPGR, New Delhi. Tissues were collected from five seed developmental stages, namely S1 [0–2 DAP (days after pollination)], S2 (3–4 DAP), S3 (5–10 DAP), S4 (11–20 DAP), and S5 (21–29 DAP) of these accessions (following Agarwal et al., 2007, 2011). Total RNA was isolated from these stages and was checked for quality as described previously (Agarwal et al., 2007; Sharma et al., 2012). To remove any contaminating DNA, RNA sample was treated with RNase-free DNase (QIAGEN) and further purified by RNeasy^®^ MinElute Cleanup Kit (QIAGEN) according to the manufacturer’s protocol. The purity and concentration of RNA samples were checked by Nanodrop 2000c Spectrophotometer (Thermo Scientific). Total cDNA was prepared from the RNA of different seed stages/tissues from IR64, for amplifying three rice *NAC* genes (*ONAC020, ONAC026*, and *ONAC023*) using the Oligo(dT) primers of Superscript^®^ III First-Strand synthesis kit (Invitrogen^TM^). The amplified PCR products generated by Phusion^®^ High-Fidelity Polymerase (New England Biolabs^®^*Inc.*) were confirmed by sequencing of at least three positive colonies per gene and extended to a maximum of 19 positive colonies for *ONAC020*. GENERUNNER V3.05^1^ was used for analyzing the various sequences. Further, a semi-quantitative PCR amplification using Phusion^®^ High fidelity Polymerase was employed for estimating the expression levels of the *trans-*spliced forms, as per the manufacturer’s protocol. Primers flanking the region of variation were used for this and the fragments were confirmed by sequencing. The amplicons were later separated on 2.5% MetaPhor^TM^ agarose (Lonza) gel and were subsequently quantified in ChemiDoc^TM^ MP imaging system by Image Lab v5.2.1 (BIORAD). In order to analyze the transcript abundance of the selected *NAC* genes, quantitative real-time PCR (Q-PCR) assay was carried out with gene-specific primers as previously described (Agarwal et al., 2007), using the Real-Time High-Capacity cDNA Reverse transcription Kit (Applied Biosystems^TM^) on 7500 Fast Real-Time PCR System (Applied Biosystems^TM^), in five seed development stages of the five accessions. The *NAC* gene expression data from three biological replicates was normalized with the rice actin gene, *ACT1* and a constant *Ct* value was used for calculation of fold changes by the 2^-Δ Δ^ *^Ct^* method.

### Promoter Analysis

To analyze the promoters of the three *NAC* genes, a 2 kb genomic region upstream to the translation start site (ATG) of each gene sequence was amplified from all five rice accessions using Phusion^®^ High-Fidelity Polymerase (New England Biolabs^®^*Inc.*) and was cloned in pJET1.2 (Thermo Scientific). High-quality sequences from a minimum of three independent colonies were analyzed for each. The promoter sequence of each *NAC* gene was analyzed in PLACE database (Higo et al., 1998) for searching *cis-*regulatory elements and these were manually compared with sequence variants discovered among accessions using Clustal X multiple alignment tool (Thompson et al., 1997).

### Analysis of SNPs amongst NAC Genes and Their Association with Seed Size/Weight

For large-scale validation and high-throughput genotyping of *NAC* gene-derived sequence variations mined among five rice accessions and to evaluate their trait association potential, the exons, introns and 2 kb URRs (upstream regulatory regions) and 1 kb DRRs (downstream regulatory regions) of three *NAC* genes were targeted for sequencing. Genomic DNA of 192 low and high grain weight rice accessions (belonging to an association panel) was resequenced employing the multiplexed amplicons resequencing method using Illumina MiSeq next-generation sequencing platform. The high-quality *NAC* gene amplicon sequence reads of each accession were mapped to pseudomolecule (version 6.0) of rice genome^2^ and the non-erroneous high-quality sequence variants (SNPs and InDels) were detected as described previously (Saxena et al., 2014; Kujur et al., 2015b). The accuracy and reliability of these identified SNPs and InDels were ascertained by comparing that with the gene promoter regions cloned and sequenced from five different accessions as mentioned above.

For genetic association analysis, phenotyping of the above mentioned 192 rice accessions was carried out. These accessions were grown for two consecutive years (as per randomized complete block design with two replications) at two diverse geographical locations (New Delhi and Tamil Nadu) of India and phenotyped for grain weight (g) trait. The weight of 1000-mature dried grains (at 10% moisture content) harvested from 10 to 15 plants of each accession with replications was estimated and diverse statistical parameters pertaining to grain weight were measured using SPSSv17.0 (Saxena et al., 2014). The population genetic structure, principal component analysis (PCA) and LD decay among the accessions using *NAC* gene-derived SNPs were determined and the association mapping was performed using CMLM (compressed mixed linear model) approach of GAPIT (Kujur et al., 2015a; Kumar et al., 2015). The relative distribution of observed and expected -log_10_(P)-value of each SNP marker-trait association was compared individually according to their derived quantile-quantile plots. The *NAC* gene-derived potential SNP loci exhibiting significant association with rice grain weight trait at highest *R*^2^ (degree of SNP marker-trait association) and lowest FDR adjusted *P*-values (threshold *P* < 1 × 10^-7^) were selected. The *NAC* gene-derived SNPs revealing high association with grain weight were validated in a traditional bi-parental F_4_ mapping population developed from inter-crossing of a low (SN with 1000 grain weight: 9.9 g) and medium (IR64: 21.4 g) grain weight parental accessions, by establishing their correlation with the phenotypes of low and high grain weight homozygous mapping individuals and were genotyped in four of each low and high grain weight homozygous mapping individuals using MALDI-TOF mass array SNP genotyping assay (Saxena et al., 2014). The genotyping data was used to constitute the haplotypes within a gene. This information was correlated with the grain weight phenotypic data of the association panel.

### Transactivation and Transrepression Assay of NAC Genes

The coding sequences of all genes and their different isoforms were fused in frame with the GAL4 DNA-binding domain of pGBKT7 vector (Clontech) and a reconstituted GAL4 TF (rGAL4, described below, manuscript submitted) for the activation and the repression assays, respectively. For the transrepression assay, the GAL4 TF of yeast was partly reconstituted by cloning the activation domain (AD) of GAL4 TF in pGBKT7 vector, which already has the binding domain (BD). AD was cloned between *NcoI* and *EcoRI* sites in the pGBKT7 vector. This construct, called as rGAL4, showed a strong transactivation property since it had both BD and AD. In line with effector-reporter assays to test the repressive ability of TFs (Ohta et al., 2001), when a repressor was fused downstream to the rGAL4 TF, it repressed the activation property of GAL4. The repression of the reporter genes depended on the strength of the repressor domain. Here, the effector is the reconstituted GAL4 fused with the protein of interest. The reporter genes are those present in the yeast strain AH109, i.e., *lacZ, HIS3*, and *ADE2*. The activity of all three reporter genes was tested for the transactivation and transrepression assays. For the assays, the ORFs of the genes were cloned between *EcoR*I and *Sal*I sites for *ONAC020* and *ONAC026*; and between *EcoR*I and *Pst*I sites for *ONAC023* in pGBKT7 and rGAL4 vectors. The constructs thus made were further transformed into yeast strain AH109 using EZ-Yeast^TM^ Transformation Kit (MP). The transformed yeast cells were selected by plating onto synthetic drop-out (SD) medium (0.667% yeast nitrogen base, 2% glucose and appropriate auxotrophic amino acid supplements) lacking tryptophan. The transactivation and transrepression properties of the constructs were determined by the differential growth of the yeast colonies on SD/-Trp/-His/-Ade media with 10 mM 3-AT in comparison with relevant controls. These were also patched onto SD/-Trp plates containing 80 mg/L of 5-bromo-4-chloro-3-indolyl-α-D-galactopyranoside (X-α-gal). These results were further confirmed by quantitative β-galactosidase enzyme (ONPG) assay as described in Clonetech^®^ Yeast protocol hand book. The β-galactosidase units were calculated for a minimum of three independent colonies for each construct, according to the formula 2000/t ^∗^(OD_420_/OD_600_), where t = time elapsed for incubation in minutes. β-galactosidase units thus obtained were further checked for their level of significance by performing an *F*-test (two-sample for variances) followed by a *t*-test with equal or unequal variance as resulted from *F*-test in Microsoft Excel^®^.

### Sub-cellular Localization and Dimerization

The sub-cellular localization of the selected *NAC* genes as well as their isoforms were predicted by analyzing their complete protein sequences using TargetP 1.1^3^, CELLO v.2.5^4^, WoLF PSORT ^5^ and Plant-mPLoc^6^. To determine the sub-cellular localization of the *NAC* genes, the full-length coding region of each gene was amplified using gene-specific primers and cloned into the Gateway^®^ entry vector pENTR^TM^/D-TOPO^®^ (Invitrogen^TM^). The resulting constructs were transferred by an LR reaction into the destination vector pSITE-3CA (Chakrabarty et al., 2007) for generation of *NAC*-*YFP* fusion constructs, under the control of a duplicated cauliflower mosaic virus (CaMV) 35S promoter. Similarly, the CDS of each gene was transferred into destination vectors for bimolecular fluorescence complementation (BiFC) assay namely, pSAT5-DEST-C(175-END)EYFP-N1 and pSAT4-DEST-N(1-174)EYFP-C1 (Tzfira et al., 2005) for N and C-terminus tagging respectively, for analyzing the protein-protein interactions. These were further transiently expressed in the onion epidermal cells by biolistics using Biolistic^®^-PDS-1000/He particle delivery system according to the manufacture’s protocol. Following overnight incubation at 28°C, the onion peels were observed in Leica TCS-SP2 Confocal Laser Scanning Microscope for YFP and mCherry signals at 514 and 594 nm, respectively. The nuclear-specific fluorochore, DAPI, was observed at 351/364 nm wavelength.

## Results and Discussion

### Three *NAC* Genes Exhibit Extreme Levels of Transcript Abundance in the Developing Rice Seed

Total transcriptome analysis has emerged as a wonderful tool for an overview of the genes controlling a particular process. Online tools and publicly available databases are a convenient source to analyze our genes or conditions of interest (Agarwal et al., 2014); and much work has been done in rice with the aim to understand and improve its yield and stability (Agarwal et al., 2016). Out of 151 genes encoding NAC TFs in rice (Nuruzzaman et al., 2010), we found nine genes [*ONAC020* (*LOC_Os01g01470*), *ONAC026* (*LOC_Os01g29840*), *ONAC023* (*LOC_Os02g12310*), *ONAC055* (*LOC_Os03g01870*), *ONAC096* (*LOC_Os07g04560*), *ONAC025* (*LOC_Os11g31330*), *ONAC127* (*LOC_Os11g31340*), *ONAC128* (*LOC_Os11g31360*), and *ONAC129* (*LOC_Os11g31380*)] to be expressing in a seed-specific manner (Supplementary Figure S1). Three out of these, *ONAC020, ONAC026*, and *ONAC023* show extremely high expression levels in our microarray data (Sharma et al., 2012), an indication of their importance to the process. *ONAC020* and *ONAC026* are closely related in the same phylogenetic branch as *CUC3*, an important gene for seed development (Supplementary Figure S2).

**FIGURE 1:**
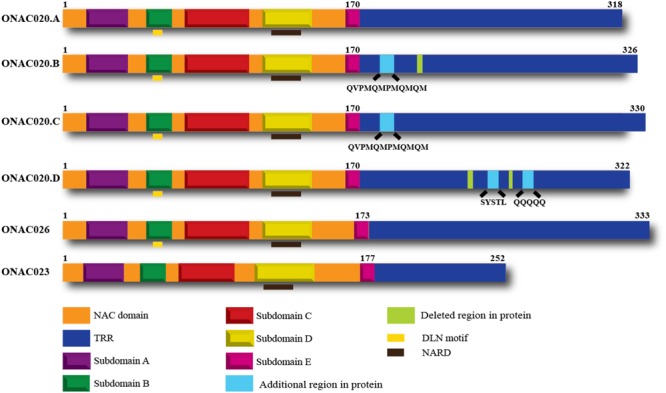
**Domains of the three NAC TFs and the different transcript fusion forms of ONAC020.** All the proteins have a NAC domain (with subdomains A–E) and a transcriptional regulation region (TRR). ONAC020 and ONAC026 have a putative DLN repressor motif in subdomain B; and all these proteins have a putative NAC repression domain (NARD) in subdomain D. The transcript fusion or *trans-*splicing between ONAC020 and 026 results in three additional proteins (ONAC020.B, 020.C, and 020.D), with insertions/deletions as marked, with respect to ONAC020.A. The color legend indicating the domains is shown at the bottom of the figure.

Hence, we decided to study the properties of these three *NAC* genes, to elucidate their similarities or differences. All these three genes have a NAC domain and a TRR. They also have the characteristic subdomains A–E and the NAC repression domain (NARD) (Hao et al., 2010). ONAC020 and ONAC026 have a DLN stretch in the B domain, which is a type of the ERF-associated amphiphilic repression/ EAR repression motif in plants (Ohta et al., 2001; Kagale et al., 2010). Preliminary analysis shows that this motif contributes to the repressive activity of these two proteins. Additionally, we identified three *trans-*spliced forms between ONAC020 and ONAC026, described further on, with changes in TRR only (**Figure 1**).

**FIGURE 2:**
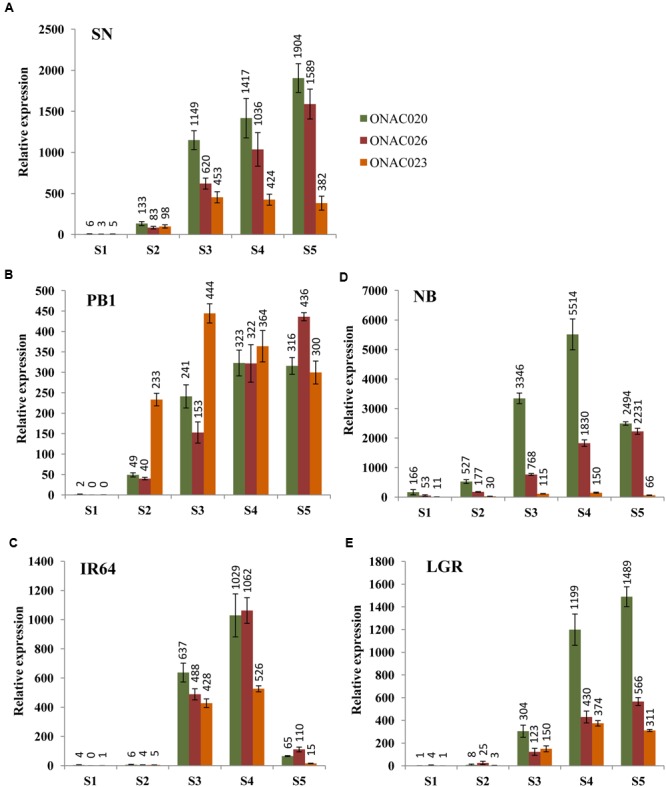
**Relative expression levels of three seed-specific *NAC* genes, namely, *ONAC020, ONAC026* and *ONAC023*, in five rice accessions with varying seed weights.** The accessions are **(A)** Sonasal (SN), **(B)** Pusa Basmati 1 (PB1), **(C)** *indica* Rice 64 (IR64), **(D)** Nipponbare (NB) and **(E)** Large Grain Rice (LGR), arranged according to increasing weights. The relative expression for each gene, in each accession has been studied in all the five stages of seed development (S1–S5), as mentioned in the color legend in **(A)**.

In order to assess their seed-specificity and relative importance, we examined the expression of the three *NAC* genes in four *indica* and one *japonica* accessions, in five stages of seed development, namely S1–S5 (Agarwal et al., 2007, 2011), by Q-PCR (**Figure 2**). SN has small sized seeds while LGR has heavier and bigger ones. PB1, IR64 and NB have medium weight grains in that order (Supplementary Figure S3). The expression levels for the genes exhibit differences in accessions. NB has the highest expression levels for *ONAC020* and *ONAC026* and lowest for *ONAC023*. The three genes also express in the S1 stage of NB, unlike other accessions (**Figure 2D**). Same is the case for small grained SN, showing a higher expression of *ONAC020* and *ONAC026*, in comparison to *ONAC023*, which is also reflected in the promoter sequence similarity analyzed further in this paper. They also show considerable expression in S2 stage (**Figure 2A**) unlike large grained LGR (**Figure 2E**). S2 represents organ initiation stage (Agarwal et al., 2011) and expression of these genes are indicative of their early role in a small-seeded variety. In PB1, the expression levels for the genes are mostly comparable in S4 and S5 stages while the same is true for S3 stage of IR64 (**Figures 2B,C**). Additionally, all the genes show a drastic drop in expression levels in the S5 stage of IR64 as compared to S3 and S4 (**Figure 2C**). In LGR, the genes increase in expression in S3, at the start of grain filling (Agarwal et al., 2011) and remain high till S5 (**Figure 2E**). Since most of the genes show low/no expression in S1, with higher levels in later stages of seed development, this possibly implies their role in one of the processes occurring during grain filling and hence, a role for grain weight. Also, these subtle variations indicate toward a degree of distinctness along with redundancy in the roles of the three seed specific *NAC* genes during rice seed development. In a similar manner, *CUC* genes involved in the initiation of ovule formation and cotyledon separation in *Arabidopsis*, show partially overlapping expression pattern and also interact amongst themselves (Goncalves et al., 2015). Since the *NAC* genes analyzed here also interact, as is shown further in the paper, the differences in their levels in the five accessions with variable seed weights, may modulate downstream seed development processes to variable extents.

**FIGURE 3:**
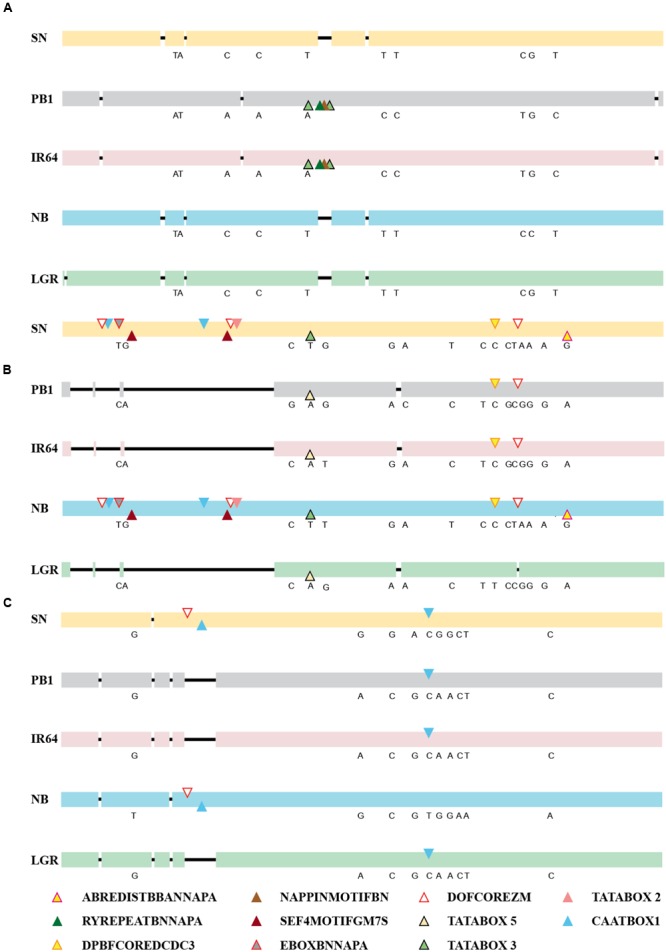
**Alignments of the promoter sequences of **(A)***ONAC020*, **(B)***ONAC026*, and **(C)***ONAC023* in five accessions of rice, namely SN, PB1, IR64, NB, and LGR.** The deletions have been shown by a black line. The SNPs among the accessions have been written in their corresponding positions (Supplementary Tables S3 and S4). Seed-specific elements with changes amongst different accessions (Supplementary Tables S5 and S6) have been marked with colored triangles, as per the color legend. The promoter elements identified on the positive strand have been marked on the upper side and those on the negative strand, on the lower side. In longer stretches of deletion, in *ONAC026* and *ONAC023*, repeatedly occurring elements in 300 and 75 bp stretches, respectively, have been represented only once by their respective triangles.

The expression of a gene is controlled directly by the presence of *cis*-elements on its promoter and their arrangement, apart from other factors. So, the 2 kb upstream region of all five genes, in the five accessions was amplified, sequenced and compared and was checked for the presence of *cis*-regulatory elements (**Figure 3**; Supplementary Table S1). For *ONAC020*, the promoter sequence is same for IR64 and PB1, while the deletions and SNPs are similar for the other three accessions. The deletion of a 30 bp stretch at 1118 bp upstream to the translation start site creates an additional RY element (CATGCA) and a Napin motif in IR64 and PB1 (**Figure 3A**). RY elements recognized by the B3 domain transcriptional activators like ABI3 and FUS3, act as important regulators of seed storage protein expression in dicots (Kawakatsu and Takaiwa, 2010). The sequence TACACAT, designated as Napin motif, is an evolutionarily conserved motif activating the expression of the seed storage protein genes in *Brassica* and soybean seeds (Jopcik et al., 2014). Correlation with the expression pattern (**Figure 2**) indicates that genotypic differences in accessions may be a controlling factor for the expression pattern of this gene. For the promoter of *ONAC026*, SN and NB have an additional stretch of 677 bp, holding multiple copies of DOFCOREZM (AAAG) and EBOXBNNAPA (CANNTG, **Figure 3B**), a probable explanation for the higher expression levels in these two accessions (**Figures 2A,D**). EBOXBNNAPA, conserved in many seed storage protein promoters is critical for directing seed-specific expression (Ellerstrom et al., 1996; Ravel et al., 2014), while DOFCOREZM is the recognition core of DOF proteins, reported to activate several storage protein genes (Yamamoto et al., 2006; Abraham et al., 2016). For *ONAC023*, the promoter sequences for SN and NB are similar, though there are SNPs amongst the two as well, and hence a similar expression pattern though levels are different. The RY element is present in multiple copies in all the promoter sequences analyzed except *ONAC023* (Supplementary Table S1), and could be a reason for its lower expression, comparatively with the other genes, in all accessions, except PB1 (**Figure 2**). On the other hand, *ONAC020*, with relatively higher expression levels, shows an abundance of the RY elements in the promoter (Supplementary Table S1). Promoter sequence analyses have revealed that the promoters of *japonica* and SN are highly similar, even though the latter is an *indica* accession and hence similar expression patterns. These two accessions are related evolutionarily (Parida et al., 2009). The overall nucleotide composition in all the sequences has been maintained due to multiple transversions. The promoter sequences have also been found to be relatively conserved closer to the start codon. Hence, the differences in expression patterns amongst the three genes in the five accessions, can be only partly accounted for by the differences in the promoter sequences. At this juncture, we can hypothesize that these may be due to varietal differences in upstream regulatory factors, amongst these accessions, which are yet to be elucidated. Additionally, chromatin modifiers have been reported to regulate the expression of different TFs involved in embryo and seedling development (Wagner, 2003; Reyes, 2006). Hence, differences in chromatin modifications amongst the five accessions may result in variation in expression in this case, a hypothesis which needs to be verified.

### *NAC* Genes Associate with Rice Grain Size/Weight Phenotype

Since SNPs were observed in the promoter sequences of the three genes amongst five accessions with variable seed weights, we aimed to elucidate any sequence variations in the entire genes, which were associated with seed size/weight character. An integrated genomic strategy by combining SNP-based association analysis, selective genotyping in bi-parental mapping population and molecular haplotyping was employed. For association mapping of grain size/weight traits in rice, targeted NGS-based resequencing of coding and non-coding (intronic and regulatory) sequence components of the three genes amongst 192 diverse low and high grain weight accessions (association panel) was performed. This identified 330 high-quality sequence variants, including 254 SNPs and 76 InDels in the three genes (Supplementary Table S2; Supplementary Figure S4). We have been able to reaffirm most of these SNPs and InDels by the individual sequencing of the promoter regions from five different accessions (Supplementary Tables S3 and S4). Almost 40% SNPs and 43.4% InDels change the *cis*-elements in the URRs (Supplementary Tables S5 and S6), some of which are essential for seed-specific expression (**Figure 3**). The 49 sequence variants mined from the exons of *NAC* genes include both 40 synonymous and non-synonymous SNPs as well as nine InDels showing frameshift mutations (Supplementary Table S2). SNPs discovered from the three *NAC* genes emphasize their utility in targeted genetic mapping and association analysis of important agronomic traits, including seed size/weight, in rice.

**FIGURE 4:**
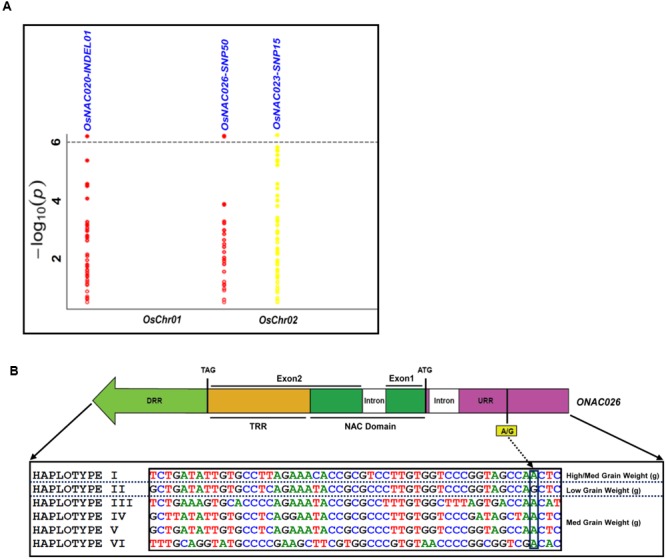
**Association of *NAC* genes with grain weight. (A)** represents significance of the sequence variants identified for the *NAC* genes, for their association with grain weight trait by the Manhattan plot. The relative density of the sequence variants identified from the *NAC* genes are taken on the X-axis with their -log_10_(P) used for scanning the significant trait associated sequence variant on the *Y*-axis. **(B)** Validation of the strong association of the InDel in the URR of *ONAC026* gene with the grain weight trait, where DRR and URR represent the downstream and upstream regulatory region respectively, ATG and TAG correspond to the translation start and stop codons respectively and TRR stands for the transcriptional regulatory region. The InDel clearly differentiates the six haplotypes into low grain weight and medium/high grain weight groups.

The use of these 254 sequence variants in population genetic structure analysis differentiates the association panel into two population clusters, POPI and POPII. The LD patterns exhibit broader LD estimates (*r*^2^: 0.23–0.89) and faster LD decay (*r*^2^ decreased half of its maximum value) nearly at 50–100 kb physical distance of rice chromosomes. This is agreed well with earlier reports on prerequisite of LD decay for effective gene-based and genome-wide association mapping studies in rice (Zhao et al., 2011; Huang et al., 2012, 2013). Hence, this LD decay is adequate enough for association mapping of SNPs with the grain size/weight trait in rice. This trait is known to follow a complex quantitative genetic inheritance pattern as observed by field phenotyping of 192 accessions in the association panel, across two diverse geographical locations, over 2 years. The trait exhibits a normal frequency distribution pattern with a broader phenotypic variation (13.5–42.7 g, mean ± SD: 26.5 ± 4.8, mean CV: 18.1%) as well as a higher heritability for grain weight (mean H^2^: 80%) (Supplementary Figures S5A,B). This necessitates essentiality of an integrated genomics-assisted breeding strategy for quantitative dissection of this complex trait. So a combinatorial strategy has been deployed involving association mapping, selective genotyping in bi-parental mapping population and molecular halotyping. Interestingly, the genetic association analysis of the *NAC* genes with the grain size/weight trait in rice identifies two regulatory SNPs located in the URRs of *ONAC026* and *ONAC023* which display remarkable association with the grain size/weight traits at a *P*-value ≤10^-6^. Also, a 7 bp regulatory InDel present in the URR of *ONAC020* is significantly associated with grain size/weight trait in rice (**Table 1**; **Figure 4A**). This is located within the *cis*-element “CACTFTPPCA1” (Supplementary Table S6), which is responsible for mesophyll-specific expression in C_4_ plants (Sharma et al., 2011). This functional regulatory InDel strongly associated with grain size/weight trait can serve as a potential candidate for marker-assisted genetic enhancement of rice. All the three natural sequence variants have diverse association potential for grain size/weight traits in rice on the basis of phenotypic variation (PVE) among the 192 accessions (**Table 1**). The sequence variants have been successfully validated in four of each low (9–12 g) and high (22–25 g) grain weight homozygous individuals of an F_4_ mapping population between IR64 and SN (Supplementary Figures S6A–C; **Table 1**). The homologs of *ONAC020* and *ONAC026* have been reported to be associated with the expression of genes encoding grain storage protein during endosperm development in wheat (Plessis et al., 2013). The above three genes were selected as target candidates for grain size/weight trait regulation by their further validation through molecular haplotyping in rice. Molecular haplotyping of *ONAC026* reveals 53 SNPs from diverse coding and non-coding (including two coding non-synonymous, three intronic, 25 DRR and 21 URR SNPs) sequence components of the gene, which form six haplotypes in the gene. These exhibit a higher degree of LD (*r*^2^ > 0.85 with *P* < 1.0 × 10^-6^) resolution (**Figure 4B**; Supplementary Figure S6D). Remarkably, the grain size/weight trait associated SNP in *ONAC026* (**Table 1**) shows strong association potential for high/medium (haplotype I) and low grain weight (haplotype II) differentiation in rice. In addition, four novel haplotypes have been identified (with diverse allelic recombination) revealing differential trait association potential for rice grain size/weight (Supplementary Figure S6D). A number of known genes underlying QTLs regulating grain size/weight have been cloned and characterized till date in rice (Zuo and Li, 2014). Also, many TFs controlling seed development have been documented (Agarwal et al., 2011). Hence, the three seed-specific genes are strongly associated with seed size/weight and are potential markers for genetic enhancement of the rice crop.

**Table 1:** *NAC* gene-derived SNPs associated with grain size/weight traits (length, width, and weight) in rice.

*OsNAC* genes	Physical positions (bp)	Associated sequence variants (SNPs/InDels)	Functional annotation	*P*-value	PVE^a^ (*R*^2^ %)
*ONAC020*-INDEL01	239034–239040	ATAC/ATACTAC	URR	3.5 × 10^-7^	34
*ONAC026*-SNP50	16722376	A/G	URR	1.2 × 10^-6^	31
*ONAC023*-SNP15	6403097	C/T	URR	2.7 × 10^-6^	27

*^a^PVE, phenotypic variation explained.*

### *ONAC020* Shows Existence of Transcript Fusion with *ONAC026*

For molecular analysis of the three *NAC* genes, they were amplified from rice cDNA, from developing seed stages. Surprisingly, *ONAC020* showed the existence of multiple forms. Analysis showed that they have arisen due to transcript fusion or *trans-*splicing with *ONAC026* (Supplementary Figures S7A,B). Rice transcriptome has been known to exhibit *trans-*splicing (Zhang G. et al., 2010), which is regulatory in nature, and increases the proteome diversity. *ONAC020* and *ONAC026* share 89% homology at the sequence level. *ONAC020.A* is the main transcript of the gene and eventually four different proteins are formed from six transcripts (A to D), with insertions/deletions in TRR only (**Figure 1**). We have named the *trans*-spliced transcripts as forms of *ONAC020* because of their higher homology with this gene. The existence of these was confirmed by semi-quantitative RT-PCR in five stages of seed development (Supplementary Figure S7C). *ONAC020.D* represents the typical form of a chimeric transcript (Dubrovina et al., 2013) between *ONAC020* and *ONAC026*. The other forms, however, seem to be the ones wherein a part of *ONAC026* has been spliced within the *ONAC020* transcript. Such transcripts may be generated due to high homology, amongst the two genes, in spliced regions. We seem to have observed a unique *trans*-splicing event, which needs to be validated further. *ONAC020* and *ONAC026* show maximum expression in the S3 and S4 stage of seed development, so do their *trans*-spliced forms. In other stages, as the levels of *ONAC020* and *ONAC026* decrease, so does the expression of the *trans-*spliced forms (Supplementary Figure S7C; Supplementary Table S7). Gene fusions, brought about by chromosomal rearrangement are reported in many neoplastic cells. Such chimeric transcripts can even result from the fusion of two transcripts without a remarkable DNA rearrangement, and can occur even in normal cells (Jividen and Li, 2014). Even though, the existence of such chimeras are not that well-established in plants, RNA sequencing experiments points to the occurrence of large number of these fusions in plants including rice (Zhang G. et al., 2010). NACs are known to have alternatively spliced forms. *OsSWN2* has an alternatively spliced form which does not cause transactivation (Yoshida et al., 2013). A splice variant of SND1, PtrSND1-A2(IR), does not transactivate or bind to DNA. It rather binds with SND1 and represses its activity (Li et al., 2012). The transcript fusion/*trans-*splicing events may vary under different accessions and needs to be looked into. Nonetheless, the variation only in the TRRs of ONAC020 leads to the speculation that the multiple forms have the same downstream targets with changes in regulatory property only, which has been examined further.

### Transcriptional Activation/Repression Ability of the Three NAC TFs Varies

**FIGURE 5:**
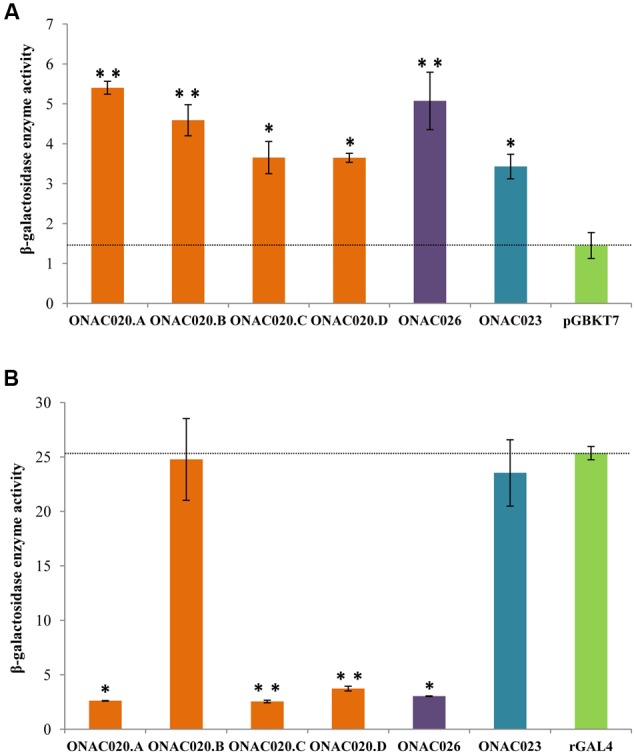
**Transcriptional ability of the NAC TFs by **(A)** transactivation and **(B)** transrepression assays of the three NAC proteins, and the proteins generated by *trans-*splicing of *ONAC020/ONAC026* (i.e., ONAC020.B–D).** The graphs show the relative β-galactosidase activity of the NAC TFs by the ONPG assay on the *Y*-axis. The vector pGBKT7 is the negative control for transactivation assays in **(A)** and the reconstituted GAL4 TF (rGAL4) is the positive control for transrepression assays in **(B)**. The standard error bars have been shown. The values which are significant with a *P*-value cut off ≤0.05 and ≤0.005 are indicated by a single and double asterisks, respectively.

Transcription regulation is a major property of TFs and most TFs harbor an activation and/or repression motif. Transactivation by the three NAC TFs was analyzed by their ability to activate the reporter genes in yeast strain AH109, in both qualitative and quantitative manners. The assay revealed that all the three proteins ONAC020.A, ONAC026, and ONAC023 can activate the reporter genes to a certain extent (**Figure 5A**; Supplementary Figures S8A,C). Since, they also have DLN and NARD motifs (**Figure 1**), their transrepression ability was also checked for, by fusion with a yeast rGAL4 TF. rGAL4 is a partly reconstituted GAL4 TF with both BD and AD domains in the same vector, in frame, which acts as a strong activator (**Figure 5B**). Since GAL4 is a strong activator in yeast, a repressor fused with the same will decrease/nullify its activation property depending on the strength of the repressor (manuscript communicated). Here, the effector is the reconstitued GAL4 fused with the protein of interest. The reporter genes are those present in the yeast strain, i.e., *lacZ, HIS3*, and *ADE2*. ONAC020.A and ONAC026 with the DLN repressor motif are able to completely abolish the activation by rGAL4 TF while ONAC023 is not a repressor (**Figure 5B**; Supplementary Figures S8B,D). Thus, ONAC020 and ONAC026 are bifunctional TFs, with low ability of activation and strong repressive activity. On the other hand, ONAC023 is a weak activator. Adding to above properties, is the fact that the presence of ONAC020 and ONAC026 cause the yeast cells to grow at a much slower rate (Supplementary Figure S8E). Again, this may be due to their strong repressive activity in yeast cells. In *Arabidopsis*, the EAR or DLN repressor proteins interact with the co-repressor TOPLESS/TPL through their “DLN” motif (Causier et al., 2012; Oh et al., 2014). TPL related proteins cause repression of target genes by interacting with histone deacetylases/HDA (Zhu et al., 2010). In yeast, repression is caused upon interaction of repressors with HDA (de Bruin et al., 2008; Lorenz et al., 2014), which bind to the promoters of G1 cyclin genes, causing repression of cell cycle (Takahata et al., 2009). It is possible that the NAC TFs causing repression are participating in a similar pathway in yeast, causing repression and hence affecting cell cycle and their growth. Since the various forms of ONAC020 differ in their TRR, they were also tested for their activation/repression properties. The three forms show slight variation in their activation ability. Interestingly, ONAC020.B, with a small deletion in the TRR shows complete abolishment of the repressive ability (**Figures 5A,B**; Supplementary Figures S8A–D). These differences in the activation/repression abilities support our above stated hypothesis of the *trans*-spliced forms having variations in regulatory properties, owing to differences in TRR only.

NAC TFs which function as repressors, possess two types of repression motifs, the NARD motif in the NAC domain and/or the DLN repressive motif. *Arabidopsis* AIF (ANTHER INDEHISCENCE FACTOR) has NARD and suppresses the jasmonic acid biosynthesis pathway to control anther dehiscence, during early flower development (Shih et al., 2014). *Arabidopsis* CBNAC is a calmodulin regulated transcriptional repressor of basal plant defense during normal growth (Kim et al., 2012). VND-INTERACTING1 (VNI1) interacts with VASCULAR-RELATED NAC-DOMAIN7 (VND7) to control the formation of *Arabidopsis* xylem vessels by acting as a repressor. VNI1 has a putative EAR domain in the C-terminal PEST motif (Yamaguchi et al., 2010). ATAF2 negatively regulates pathogenesis related genes (Delessert et al., 2005). Just like the genes examined here, few NAC TFs have been shown to possess both activation and repression domains and hence act as bifunctional TFs, such as GmNAC20 (Hao et al., 2011) and VNI2 (Yang et al., 2011). Apart from NACs, *Arabidopsis* WUSCHEL and Histone-Like NF-Y are bifunctional TFs (Ceribelli et al., 2008; Ikeda et al., 2009). The variation in activation/repression properties and expression patterns of the three NAC TFs provides fuel to our theory of the genes having overlapping as well as independent functions during rice seed development. Moreover, the variations in the properties of the *trans-*spliced forms of the genes further emphasizes their role as competitors (Reddy et al., 2013).

### The NAC TFs Localize Differently and Heterodimerize

**FIGURE 6:**
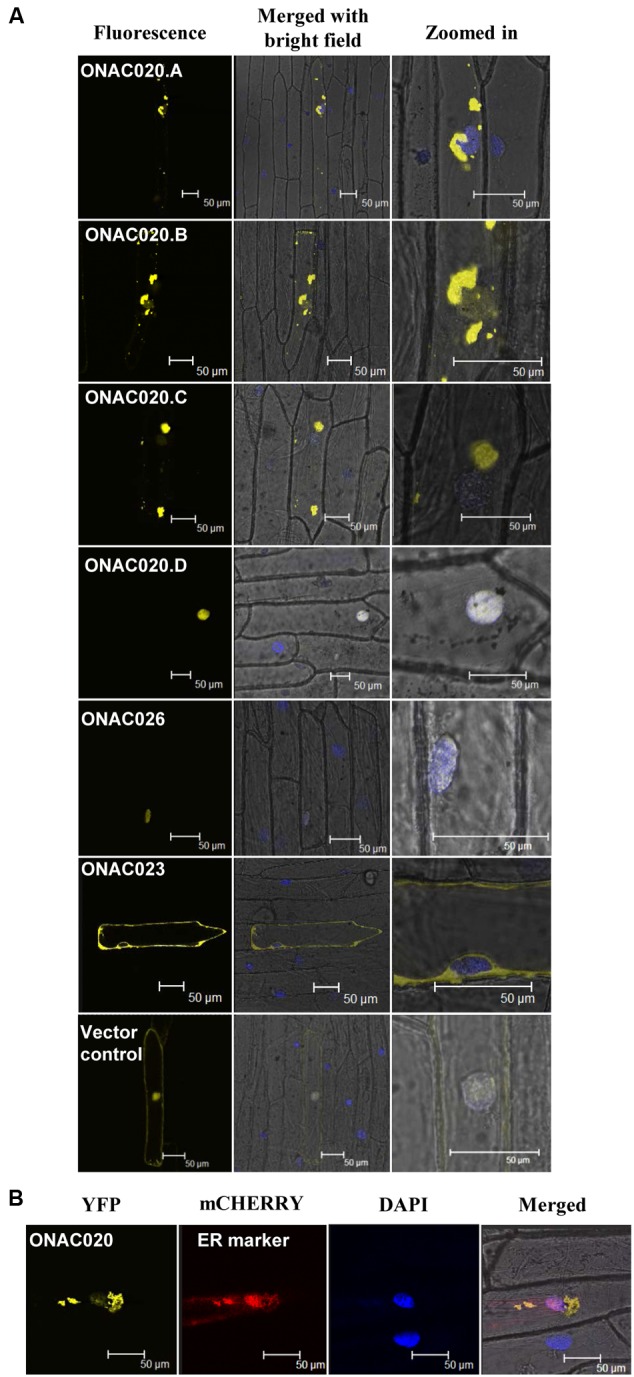
**Subcellular localization of NAC-YFP fusion proteins. (A)** represents the localization images of the three main NAC TFs and the transcript fusion forms of ONAC020. The left column indicates the localization image of the YFP fusion protein at 514 nm. The middle column is the merger of bright field, fluorescence image and DAPI stained nucleus while the right column shows an enlarged image. **(B)** represents the co-localization of ONAC020 with endoplasmic reticulum (ER) marker. The protein names have been mentioned and the scale bar indicates 50 μm.

For TFs to carry out their function, they need to be localized to the nucleus, either independently or in conjunction with other TFs. NAC TFs have been predicted to have transmembrane domains, nuclear localization signals/NLS and nuclear export signals/NES (Olsen et al., 2005). The three NAC TFs analyzed here have been predicted to possess various localization signals (Supplementary Table S8). Their fusion products with YFP show that only ONAC026 is completely nuclear localized in onion peel cells. ONAC023 is localized in the cytoplasm (**Figure 6A**) and ONAC020.A in the endoplasmic reticulum (**Figures 6A,B**). Amongst the various forms arising due to *trans-*splicing, ONAC020.B and ONAC020.C are localized like the main form. Interestingly, ONAC020.D goes to the nucleus (**Figure 6A**). Splicing of membrane bound TFs, such as ER-membrane bound bZIP60 leads to a functional nuclear-targeted form (Seo, 2014) as is the case with ONAC020.D. CELLO and Plant-mPLoc predict nuclear localization of all six proteins (ONAC020, ONAC026, three *trans*-spliced transcripts and ONAC023; Supplementary Table S8). However, only ONAC020.D and ONAC026 were localized in the nucleus (**Figure 6**). Only sometimes localization predictions are not validated (Chaturvedi et al., 2014). The cause for this variation can only be hypothesized here. The protein conformation may not be exposing the nuclear localization signal efficiently for such results. Additionally, ER is required for protein trafficking (Chen et al., 2012) and the proteins might have accumulated there. Amongst the other NACs, ATAF1, a founding member of the NAC family, is localized to the nucleus (Lu et al., 2007). NTL4 is processed and localized to the nucleus only upon heat stress (Lee et al., 2014). A few NAC TFs localize to organelles other than nucleus. MaNAC6 in banana gets localized to the cell membrane, cytoplasm, and nucleus unlike the nuclear localized MaNAC1–5 (Shan et al., 2012). ANAC of *Arabidopsis* gets localized in both the nucleus and cytoplasm. It interacts with two RING-H2 domain proteins, both of which also show similar localization patterns. The authors hypothesize that the proteins may interact in the nucleus and then be exported outside (Greve et al., 2003). Apart from this, a significant feature of many NAC TFs is the transmembrane domain (TM). ANAC017 is localized to the ER. Subsequent to the cleavage of the TM domain, it gets localized in the nucleus and mediates stress resistance (Ng et al., 2013). Similarly, membrane bound *Arabidopsis* NTM1 and bZIP are activated by proteolytic cleavage leading to a functional nuclear targeted form (Kim et al., 2007; Iwata et al., 2008).

**FIGURE 7:**
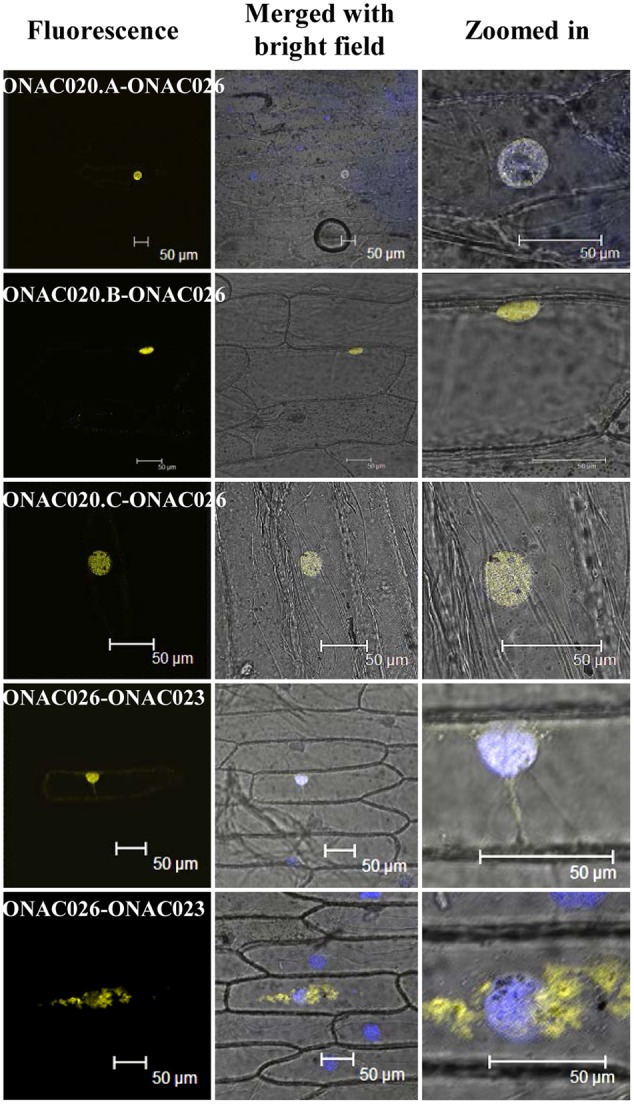
**Interactions amongst the NAC proteins by BiFC.** The left column indicates the localization image of the reconstituted YFP protein at 514 nm. The middle column is the merger of bright field and fluorescence images while the right column shows an enlarged image of the nucleus. The nucleus has been stained by DAPI dye. The interacting protein names have been mentioned and the scale bar indicates 50 μm.

Since only ONAC020.D and ONAC026 are nuclear localized, the others may do so either by proteolytic cleavage during seed development or by heterodimerization. The same was proven when it was found that ONAC026 interacts with three ER localized forms of ONAC020 (ie. ONAC020.A, ONAC020.B, and ONAC020.C), and all the dimers are directed to the nucleus in BiFC experiments. Additionally, ONAC026 and ONAC023 also interact, and the dimers are found in either the nucleus or ER, in an equal number of experiments (**Figure 7**). Surprisingly, ONAC020.D does not interact with ONAC026. Neither does it interact with the other three *trans-*spliced forms of ONAC020. For TFs to activate/supress target genes, they have to be targeted to the nucleus. Often, TFs dimerize with others having a NLS and are thus nuclear localized (Withers et al., 2012; Nayar et al., 2014). Such is the case with the three *trans-*spliced forms of ONAC020 and ONAC023, which interact with ONAC026 (**Figure 7**). However, ONAC023–026 dimers were found in the ER as well. Sometimes, TFs are localized to ER, and get nuclear localized upon proteolytic cleavage (De Clercq et al., 2013; Hofmann, 2013; Ng et al., 2013). This may be because ER serves as the port of entry of many proteins which are destined to other organelles along with the ER resident proteins. It acts as the site of folding and maturation of proteins (Galili et al., 1998). Additionally, it is possible that other interacting partners are required for the complex to completely localize to the nucleus, which can be proven only by further experimentation.

A protein lacking a few functional domains is called as an interfering protein/small interfering peptide (Seo et al., 2011). Isoforms of a TF may lack one of the functional domains, keeping other functions intact and hence behave as dominant-negative regulators or competitors (Reddy et al., 2013) and hence ONAC020.D may function as a dominant-negative regulator. Also, ONAC020.A and its forms do not interact with ONAC023 in our BiFC experiments. Amongst known NAC interactors, GmNAC30 and GmNAC81, which are bifunctional TFs interact in the nucleus and bind to the promoter of *VPE* to integrate various stress responses (Mendes et al., 2013). ANAC096 interacts with ABF2 and ABF4 to impart abiotic stress resistance (Xu et al., 2013). Regulatory networks are an important aspect of seed development, and often involve formation of homo/heterodimers between TFs. In barley, HvVP1 interacts with HvGAMYB and BPBF and represses their DNA-binding activity (Abraham et al., 2016). Maize OPAQUE2 dimerizes with MADS47 and enhances its activation of zein genes (Qiao et al., 2016). Similarly, the well-known LAFL network of seed maturation involves formation of many hetero and homodimers (Agarwal et al., 2011; Jia et al., 2014). Hence, the observation that ONAC020 and ONAC023 interact with ONAC026 is important. Moreover, the localization of these heteromers to the nucleus, further signifies the transcriptional role of these dimers and makes for an interesting study. Hence, the seed-specific NAC TFs interact amongst each other and subsequently enter the nucleus to bring about their function.

### Role-Play amongst NAC Members during the Progression of Rice Seed Development

In spite of huge efforts, very limited number of potential robust genes/QTLs have been deployed in marker-assisted genetic improvement of rice and limited information is available about their functional aspects. Here, we have identified three seed-specific NAC TFs, with variation in their expression patterns in five different rice accessions over a range of seed size/weight trait, which is controlled by sequence variations in the promoter regions to a certain extent. All the three genes *ONAC020, 026*, and *023* are significantly associated with seed size/weight trait in rice, with the associated sequence variants in the URRs. *ONAC020* and *026* exhibit *trans-*splicing. ONAC026, a strong repressor, dimerizes with ONAC020.A, ONC020.B, ONAC020.C, and ONAC023 and these heterodimers are nuclear localized. Hence, a complex is formed amongst ONAC020, 026, and 023. It is highly probable that the repression/ activation levels of the complex is an overall combination of all the components, which may vary amongst the various stages of seed development amongst accessions, as indicated by expression pattern and sequence variations. For example, the expression pattern for *ONAC020* and *ONAC026* is similar for an accession, though the levels may be different and would suggest the significance of these interactions in controlling seed development. In conclusion, it is proposed here that in the five rice accessions, there is interaction and subsequent nuclear localization amongst three seed-specific NAC TFs, with variable levels of activation and repression. The fusion forms act as competitors or interfering peptides. The genes may be a part of a bigger network controlling seed size/weight in these accessions, based on their differential expression patterns and association analysis. Functional characterization of genotypes/transgenic plants with altered expression of these genes or with alleles controlling high seed size/weight will provide further insight about the same. These genes can be useful for rapid quantitative dissection of complex grain size/weight trait in rice to eventually accelerate the development of rice cultivars with high grain weight and yield.

## Accession Numbers

The accession IDs at NCBI BankIt database for cDNA sequences from IR64 are KX953272 for ONAC020.A, KX953273 for ONAC020.B, KX953274 for ONAC020.C, KX953275 for ONAC020.D, KX953276 for ONAC020.E, KX953277 for ONAC020.F, KX953278 for ONAC026, and KX953279 for ONAC023. The NCBI BankIt accession IDs for 2kb upstream regions of ONAC020 are KX953280 from SN, KX953281 from LGR, KX953282 from IR64 and KX953283 from PB1; for ONAC026 are KX953284 from SN, KX953285 from LGR, KX953286 from IR64, KX953287 from PB1; for ONAC023 are KX953288 from SN, KX953289 from LGR, KX953290 from IR64 and KX953291 from PB1.

## Author Contributions

IM performed the experiments. SD performed the transrepression assay of the multiple forms of the genes. AM collected seeds from different accessions and isolated RNA. PA and IM wrote the manuscript and prepared the figures. PA conceptualized and supervised the experiments. All authors have reviewed the manuscript.

## Conflict of Interest Statement

The authors declare that the research was conducted in the absence of any commercial or financial relationships that could be construed as a potential conflict of interest.

## Footnotes

1 http://generunner.net/

2 http://rice.plantbiology.msu.edu

3 http://www.cbs.dtu.dk/services/TargetP/

4 http://cello.life.nctu.edu.tw/

5 http://www.genscript.com/psort/wolf_psort.html

6 http://www.csbio.sjtu.edu.cn/bioinf/plant-multi/
