# Supplementary material for: Three Rice NAC Transcription Factors Heteromerize and Are Associated with Seed Size
Source: Front Plant Sci. 2016 Nov 7;7:1638. doi: 10.3389/fpls.2016.01638 (PMC5098391; doi:10.3389/fpls.2016.01638)
Supplement: Supplementary file 1 [file Table_1.pdf]

Supplementary Table S1: Comparison of *cis* -elements predicted within the *NAC* genes

***cis*- element name**	***ONAC020***	***ONAC026***	***ONAC023***
-10PEHVPSBD	2	4	2
2SSEEDPROTBANAPA	1	1	1
-300CORE	0	0	1
-300ELEMENT	0	0	3
5659BOXLELAT5659	0	0	2
AACACOREOSGLUB1	1	1	0
ABREDISTBBNNAPA	1	1	0
ABREATRD22	0	0	0
ABRELATERD1	3	5	1
ABRERATCAL	3	2	2
ACGTATERD1	6	8	10
ACGTCBOX	0	0	2
ACGTOSGLUB1	0	2	0
AMYBOX1	0	0	0
AMYBOX2	3	1	0
ANAERO1CONSENSUS	5	3	5
ANAERO3CONSENSUS	0	0	1
ARFAT	1	1	1
ARR1AT	17	33	26
ASF1MOTIFCAMV	5	1	1
BIHD1OS	3	4	6
BOXCPSAS1	0	0	1
BOXIINTPATPB	2	3	3
BOXLCOREDCPAL	1	1	0
BP5OSWX	1	0	0
CAATBOX1	16	18	27
CACGTGMOTIF	0	2	0
CACGCAATGMGH3	0	0	1
CACTFTPPCA1	30	31	15
CANBNNAPA	1	1	3
CARGATCONSENSUS	0	0	0
CAREOSREP1	0	0	3
CARGCW8GAT	6	4	6
CATATGGMSAUR	10	0	2
CBFHV	0	0	4
CCA1ATLHCB1	1	6	1
CCAATBOX1	3	4	6
CIACADIANLELHC	0	0	0
CDA1ATCAB2	0	0	0
CGACGOSAMY3	0	0	4
CGCGBOXAT	8	2	2
CIACADIANLELHC	1	1	1
CPBCSPOR	0	1	0
CRTDREHVCBF2	0	0	2

**Supplementary Table S1: Comparison of *cis* -elements predicted within the *NAC* genes**			
***cis*- element name**	***ONAC020***	***ONAC026***	***ONAC023***
CURECORECR	8	8	20
DOFCOREZM	15	27	37
DPBFCOREDCDC3	9	6	6
DRE1COREZMRAB17	0	0	0
DRECRTCOREAT	0	0	0
E2FCONSENSUS	0	0	1
EBOXBNNAPA	30	18	22
EECCRCAH1	3	6	2
EMHVCHORD	0	0	0
ERELEE4	1	0	1
EVENINGAT	0	0	0
GAREAT	1	1	0
GARE1OSREP1	0	0	0
GATABOX	34	31	21
GCN4OSGLUB1	0	0	0
GT1CONSENSUS	8	15	19
GT1CORE	3	2	0
GT1GMSCAM4	1	6	8
GTGANTG10	16	18	18
HEXAT	1	1	0
HEXAMERATH4	0	0	1
HEXMOTIFTAH3H4	2	1	1
IBOX	2	2	2
IBOXCORE	4	3	6
IBOXCORENT	0	2	2
INRNTPSADB	3	1	4
IRO2OS	0	0	0
INTRONLOWER	2	1	1
LEAFYATAG	1	1	1
LECPLEACS2	1	0	0
LTRE1HVBLT49	1	1	1
LTREATLTI78	0	0	0
LTRECOREATCOR15	0	0	0
MARTBOX	0	0	4
MNF1ZMPPC1	0	0	0
MYB1AT	3	2	1
MYB2AT	1	0	0
MYB1LEPR	0	0	1
MYB2CONSENSUSAT	2	2	1
MYBATRD22	0	0	0
MYBCORE	3	2	3
MYBCOREATCYCB1	1	2	4
MYBGAHV	0	0	0
MYBPLANT	0	0	0

**Supplementary Table S1: Comparison of *cis*-elements predicted within the *NAC* genes**			
***cis*- element name**	***ONAC020***	***ONAC026***	***ONAC023***
MYBPZM	2	1	2
MYBST1	8	4	7
MYCATERD1	6	2	1
MYCATRD22	0	2	1
MYCCONSENSUSAT	30	18	22
NAPINMOTIFBN	0	1	2
NODCON1GM	2	9	4
NODCON2GM	2	1	7
NTBBF1ARROLB	2	2	0
OSE1ROOTNODULE	2	9	4
OSE2ROOTNODULE	2	1	7
P1BS	6	2	6
PALBOXPPC	0	0	0
POLASIG1	2	7	4
POLASIG2	2	4	3
POLASIG3	4	2	4
POLLEN1LELAT52	8	13	15
PREATPRODH	0	0	3
PRECONSCRHSP70A	5	1	2
PROLAMINBOXOSGLUB1	1	1	1
PYRIMIDINEBOXHVEPB1	0	1	0
PYRIMIDINEBOXOSRAMY1A	1	0	2
QARBNEXTA	0	0	0
RAV1AAT	6	3	4
RAV1BAT	0	0	1
RBCSCONSENSUS	0	3	2
REALPHALGLHCB21	1	2	2
REBETALGLHCB21	2	0	0
RHERPATEXPA7	2	2	2
RNFG1OS	1	0	0
ROOTMOTIFTAPOX1	10	9	7
RYREPEATBNNAPA	13	3	0
RYREPEATGMGY2	8	2	0
RYREPEATLEGUMINBOX	8	2	0
RYREPEATVFLEB4	4	2	0
S1FBOXSORPS1L21	2	3	2
SEBFCONSSTPR10A	1	1	2
SEF1MOTIF	0	0	0
SEF3MOTIFGM	2	1	1
SEF4MOTIFGM7S	2	9	4
SITEIIATCYTC	0	1	0
SITEIIBOSPCNA	0	0	0
SORLIP1AT	2	2	1
SORLIP2AT	0	0	0

**Supplementary Table S1: Comparison of *cis* -elements predicted within the *NAC* genes**			
***cis-* element name**	***ONAC020***	***ONAC026***	***ONAC023***
SORLIP5AT	0	6	0
SORLREP3AT	2	1	1
SP8BFIBSP8BIB	1	0	0
SREATMSD	1	0	2
SURECOREATSULTR11	3	1	2
SV40COREENHAN	0	0	1
T/GBOXATPIN2	1	0	1
TAAAGSTKST1	7	9	4
TATABOX1	0	0	2
TATABOX2	1	2	2
TATABOX3	0	3	2
TATABOX4	2	2	1
TATABOX5	2	0	2
TATABOXOSPAL	1	0	1
TATAPVTRNALEU	0	0	0
TATCCACHVAL21	0	0	1
TATCCAOSAMY	3	1	4
TATCCAYMOTIFOSRAMY3D	3	1	1
TBOXATGAPB	1	1	2
TGACGTVMAMY	2	1	1
TGTCACACMCUCUMISIN	1	0	1
TRANSINITDICOTS	1	1	1
TRANSINITMONOCOTS	1	1	2
UPRMOTIFIAT	1	1	0
WBOXATNPR1	0	0	0
WBOXHVISO1	4	3	2
WBOXNTCHN48	2	2	3
WBOXNTERF3	4	3	4
WRKY71OS	12	8	11
WUSATAg	1	0	0
XYLAT	0	0	1

Note: The highlighted elements represent the ones needed for seed-specific expression.

Supplementary Table S2: Distribution and structural annotation of the sequence variants (SNPs and InDels) discovered from the three rice *NAC* genes

NAC genes	Number (%) of genic sequence polymorphism discovered		Upstream regulatory regions (URRs)		Downstream regulatory regions (DRRs)		Introns		Exons		
	**SNPs**	**InDels**	**SNPs**	**InDels**	**SNPs**	**InDels**	**SNPs**	**InDels**	**Non-synonymous SNPs**	**Synonymous SNPs**	**InDels**
ONAC020	65 (25.6)	30 (39.5)	31	16	26	5	2	4	4	2	5
ONAC026	53 (20.9)	15 (19.7)	21	5	25	4	3	3	2	2	3
ONAC023	136 (53.5)	31(40.8)	49	12	33	11	24	7	13	17	1
**Total**	**254**	**76**	**101 (47.2)**	**33 (49.2)**	**84 (39.3)**	**20 (29.9)**	**29 (13.5)**	**14 (20.9)**	**19 (47.5)**	**21 (52.5)**	**9**
**Grand Total**	**330**		**134 (40.6)**		**104 (31.5)**		**43 (13.0)**		**49 (14.9)**		

Supplementary Table S3: SNP validation in the promoter regions sequenced from five accessions							
**NAC gene IDs**	**Upstream promoter position**	**Sonasal**	**Pusa Basmati1**	**IR64**	**Nipponbare**	**LGR**	**Physical position (bp)**
ONAC020	-1443	G	T	T	G	G	239464
ONAC020	-1326	C	A	A	C	C	239581
ONAC020	-964	T	C	C	T	T	239943
ONAC020	-894	T	C	C	T	T	240013
ONAC020	-469	C	T	T	C	C	240438
ONAC020	-465	G	G	G	C	G	240442
ONAC020	-381	T	C	C	T	T	240526
ONAC026	-1667	C	T	T	C	T	16721658
ONAC026	-1601	T	C	C	T	C	16721724
ONAC026	-1550	T	C	C	T	C	16721775
ONAC026	-1504	T	C	C	T	C	16721821
ONAC026	-1481	A	G	G	A	G	16721844
ONAC026	-1478	G	C	C	G	G	NA
ONAC026	-1434	G	G	G	G	A	16721891
ONAC026	-1397	G	A	A	G	A	16721928
ONAC026	-1243	A	G	G	A	G	16722082
ONAC026	-1089	C	C	C	C	T	16722236
ONAC026	-850	C	C	A	A	C	NA
ONAC026	-821	A	T	T	A	T	16722504
ONAC026	-757	G	C	G	G	G	NA
ONAC023	-1676	G	G	G	T	G	6402406
ONAC023	-923	G	A	A	G	A	6403159
ONAC023	-871	G	C	C	C	C	6403211
ONAC023	-741	A	G	G	G	G	6403341
ONAC023	-710	C	C	C	T	C	6403372
ONAC023	-666	G	A	A	G	A	6403416
ONAC023	-633	G	A	A	G	A	6403449
ONAC023	-597	C	C	C	A	C	6403485
ONAC023	-589	T	T	T	A	T	6403493
ONAC023	-293	C	C	C	A	C	6403789

NA denotes those SNPs which are present in the individual sequencing of the promoter regions, but are not validated in the resequencing data.

Supplementary Table S4: Validation of InDels in the promoter regions sequenced from five accessions							
**NAC gene IDs**	**Upstream promoter position**	**Sonasal**	**Pusa Basmati1**	**IR64**	**Nipponbare**	**LGR**	**Physical position (bp)**
ONAC020	-1979	A	A	A	A	_	NA
ONAC020	-1867	CTA	_	_	CTA	CTA	239040
ONAC020	-1644	_	ATAG	ATAG	_	_	239263
ONAC020	-1578	_	TA	TA	_	_	239329
ONAC020	-1402	C	_	_	C	C	239505
ONAC020	-1125	_	ATGGCAAAATGTGTAAATTAATTAATATGG	ATGGCAAAATGTGTAAATTAATTAATATGG	_	_	239782
ONAC020	-1007	_	AT	AT	_	_	239900
ONAC020	-46	CTATATATG	_	_	CTATATATG	CTATATATG	240861
ONAC026	-1495	T	T	T	T	_	NA
ONAC026	-1128	GAG	_	_	GAG	_	16722197
ONAC026	1308- ~1970	PRESENT	ABSENT	ABSENT	PRESENT	ABSENT	NA
ONAC023	-1789	T	_	_	_	_	NA
ONAC023	-1615	_	_	_	T	_	6402467
ONAC023	-1549	T	_	_	_	_	NA
ONAC023	-1501	Additional 75 bp fragment	_	_	Additional 75 bp fragment	_	NA

NA denotes those InDels which are present in the individual sequencing of the promoter regions, but are not validated in the resequencing data.

Supplementary Table S5: Details of SNPs within the selected *NAC* genes. The *cis*- element changes in URR because of an SNP have also been mentioned.

SNP IDs	Genes	Physical position (bp)	SNPs	Structural annotation	*Cis* -elements predicted in that region	Upstream promoter position
ONAC020-SNP01	LOC_Os01g01470	238914	C/T	URR	PRECONSCRHSP70A	-1993
ONAC020-SNP02	LOC_Os01g01470	238977	G/C	URR	REBETALGLHCB21, MYBST1	-1930
ONAC020-SNP03	LOC_Os01g01470	239059	T/C	URR	REBETALGLHCB21, MYBST1, GATABOX	-1848
ONAC020-SNP04	LOC_Os01g01470	239275	A/G	URR	CURECORECR	-1632
ONAC020-SNP05	LOC_Os01g01470	239464	T/G	URR	SURECOREATSULTR11	-1443
ONAC020-SNP06	LOC_Os01g01470	239559	G/C	URR	NA	-1348
ONAC020-SNP07	LOC_Os01g01470	239581	A/C	URR	INTRONLOWER	-1326
ONAC020-SNP08	LOC_Os01g01470	239741	A/T	URR	NA	-1166
ONAC020-SNP09	LOC_Os01g01470	239763	C/T	URR	NA	-1144
ONAC020-SNP10	LOC_Os01g01470	239943	C/T	URR	NA	-964
ONAC020-SNP11	LOC_Os01g01470	239946	G/A	URR	NA	-961
ONAC020-SNP12	LOC_Os01g01470	240008	G/A	URR	NA	-899
ONAC020-SNP13	LOC_Os01g01470	240013	T/C	URR	DOFCOREZM, GT1CONSENSUS, GT1GMSCAM4	-894
ONAC020-SNP14	LOC_Os01g01470	240059	A/T	URR	NA	-848
ONAC020-SNP15	LOC_Os01g01470	240071	A/C	URR	NA	-836
ONAC020-SNP16	LOC_Os01g01470	240226	A/G	URR	CACTFTPPCA1, TBOXATGAPB	-681
ONAC020-SNP17	LOC_Os01g01470	240413	C/T	URR	NA	-494
ONAC020-SNP18	LOC_Os01g01470	240425	T/C	URR	NA	-482
ONAC020-SNP19	LOC_Os01g01470	240438	T/C	URR	NA	-469
ONAC020-SNP20	LOC_Os01g01470	240442	G/C	URR	ASF1MOTIFCAMV, WRKY71OS	-465
ONAC020-SNP21	LOC_Os01g01470	240479	C/T	URR	NA	-428
ONAC020-SNP22	LOC_Os01g01470	240485	C/T	URR	CAATBOX1, EBOXBNNAPA, MYCCONSENSUSAT	-422
ONAC020-SNP23	LOC_Os01g01470	240512	G/A	URR	NA	-395
ONAC020-SNP24	LOC_Os01g01470	240524	G/A	URR	MYB2CONSENSUSAT, MYBCORE, MYBCOREATCYCB1, MYBPZM	-383
ONAC020-SNP25	LOC_Os01g01470	240526	C/T	URR	MYBPZM	-381
ONAC020-SNP26	LOC_Os01g01470	240557	G/A	URR	ABREDISTBBNNAPA	-350

Supplementary Table S5: Details of SNPs within the selected *NAC* genes. The *cis-* element changes in URR because of an SNP have also been mentioned.						
**SNP IDs**	**Genes**	**Physical position (bp)**	**SNPs**	**Structural annotation**	***Cis*-elements predicted in that region**	**Upstream promoter position**
ONAC020-SNP27	LOC_Os01g01470	240571	G/A	URR	NA	-336
ONAC020-SNP28	LOC_Os01g01470	240873	C/T	URR	NA	-34
ONAC020-SNP29	LOC_Os01g01470	240893	T/C	URR	CACTFTPPCA1	-14
ONAC020-SNP30	LOC_Os01g01470	240900	T/C	URR	NA	-7
ONAC020-SNP31	LOC_Os01g01470	240906	G/A	URR	NA	-1
ONAC020-SNP32	LOC_Os01g01470	241121	A/G	INTRON		
ONAC020-SNP33	LOC_Os01g01470	241181	A/G	INTRON		
ONAC020-SNP34	LOC_Os01g01470	241339	A/G	CDS-Non-CDS-Synonymous SNP		
ONAC020-SNP35	LOC_Os01g01470	241656	C/A	CDS-Synonymous SNP		
ONAC020-SNP36	LOC_Os01g01470	241671	G/A	CDS-Synonymous SNP		
ONAC020-SNP37	LOC_Os01g01470	241742	T/C	CDS-Non-CDS-Synonymous SNP		
ONAC020-SNP38	LOC_Os01g01470	241787	A/C	CDS-Non-CDS-Synonymous SNP		
ONAC020-SNP39	LOC_Os01g01470	241949	C/A	CDS-Non-CDS-Synonymous SNP		
ONAC020-SNP40	LOC_Os01g01470	242006	A/G	DRR		
ONAC020-SNP41	LOC_Os01g01470	242062	T/C	DRR		
ONAC020-SNP42	LOC_Os01g01470	242072	T/C	DRR		
ONAC020-SNP43	LOC_Os01g01470	242073	T/A	DRR		
ONAC020-SNP44	LOC_Os01g01470	242219	G/C	DRR		
ONAC020-SNP45	LOC_Os01g01470	242316	G/A	DRR		
ONAC020-SNP46	LOC_Os01g01470	242393	T/C	DRR		
ONAC020-SNP47	LOC_Os01g01470	242559	T/C	DRR		
ONAC020-SNP48	LOC_Os01g01470	242621	A/G	DRR		
ONAC020-SNP49	LOC_Os01g01470	242867	T/C	DRR		

**Supplementary Table S5: Details of SNPs within the selected *NAC* genes. The *cis*- element changes in URR because of an SNP have also been mentioned.**						
**SNP IDs**	**Genes**	**Physical position (bp)**	**SNPs**	**Structural annotation**	***Cis*-elements predicted in that region**	**Upstream promoter position**
ONAC020-SNP50	LOC_Os01g01470	242879	A/G	DRR		
ONAC020-SNP51	LOC_Os01g01470	242883	T/A	DRR		
ONAC020-SNP52	LOC_Os01g01470	242923	G/A	DRR		
ONAC020-SNP53	LOC_Os01g01470	242931	T/C	DRR		
ONAC020-SNP54	LOC_Os01g01470	242995	T/A	DRR		
ONAC020-SNP55	LOC_Os01g01470	243041	G/A	DRR		
ONAC020-SNP56	LOC_Os01g01470	243044	T/C	DRR		
ONAC020-SNP57	LOC_Os01g01470	243111	G/A	DRR		
ONAC020-SNP58	LOC_Os01g01470	243145	T/C	DRR		
ONAC020-SNP59	LOC_Os01g01470	243153	C/T	DRR		
ONAC020-SNP60	LOC_Os01g01470	243154	C/T	DRR		
ONAC020-SNP61	LOC_Os01g01470	243212	C/A	DRR		
ONAC020-SNP62	LOC_Os01g01470	243311	A/C	DRR		
ONAC020-SNP63	LOC_Os01g01470	243334	G/A	DRR		
ONAC020-SNP64	LOC_Os01g01470	243420	T/C	DRR		
ONAC020-SNP65	LOC_Os01g01470	243462	C/A	DRR		
ONAC026-SNP01	LOC_Os01g29840	16718568	T/G	DRR		
ONAC026-SNP02	LOC_Os01g29840	16718619	C/T	DRR		
ONAC026-SNP03	LOC_Os01g29840	16718676	T/A	DRR		
ONAC026-SNP04	LOC_Os01g29840	16718757	G/T	DRR		
ONAC026-SNP05	LOC_Os01g29840	16718783	A/C	DRR		
ONAC026-SNP06	LOC_Os01g29840	16718839	T/A	DRR		
ONAC026-SNP07	LOC_Os01g29840	16718941	A/G	DRR		
ONAC026-SNP08	LOC_Os01g29840	16719055	T/G	DRR		
ONAC026-SNP09	LOC_Os01g29840	16719093	T/G	DRR		
ONAC026-SNP10	LOC_Os01g29840	16719102	G/A	DRR		
ONAC026-SNP11	LOC_Os01g29840	16719119	T/C	DRR		
ONAC026-SNP12	LOC_Os01g29840	16719130	G/A	DRR		

Supplementary Table S5: Details of SNPs within the selected *NAC* genes. The *cis*- element changes in URR because of an SNP have also been mentioned.

SNP IDs	Genes	Physical position (bp)	SNPs	Structural annotation	*Cis* -elements predicted in that region	Upstream promoter position
ONAC026-SNP13	LOC_Os01g29840	16719154	C/T	DRR		
ONAC026-SNP14	LOC_Os01g29840	16719169	C/A	DRR		
ONAC026-SNP15	LOC_Os01g29840	16719310	T/C	DRR		
ONAC026-SNP16	LOC_Os01g29840	16719329	C/T	DRR		
ONAC026-SNP17	LOC_Os01g29840	16719456	A/G	DRR		
ONAC026-SNP18	LOC_Os01g29840	16719688	G/A	DRR		
ONAC026-SNP19	LOC_Os01g29840	16719700	A/G	DRR		
ONAC026-SNP20	LOC_Os01g29840	16719797	A/G	DRR		
ONAC026-SNP21	LOC_Os01g29840	16719799	A/C	DRR		
ONAC026-SNP22	LOC_Os01g29840	16720155	T/C	DRR		
ONAC026-SNP23	LOC_Os01g29840	16720174	A/T	DRR		
ONAC026-SNP24	LOC_Os01g29840	16720185	C/T	DRR		
ONAC026-SNP25	LOC_Os01g29840	16720196	C/G	DRR		
ONAC026-SNP26	LOC_Os01g29840	16720293	G/T	CDS-Non-Synonymous SNP		
ONAC026-SNP27	LOC_Os01g29840	16720395	C/G	CDS-Non-Synonymous SNP		
ONAC026-SNP28	LOC_Os01g29840	16720512	G/A	CDS-Synonymous SNP		
ONAC026-SNP29	LOC_Os01g29840	16720770	C/T	CDS-Synonymous SNP		
ONAC026-SNP30	LOC_Os01g29840	16721048	C/T	INTRON		
ONAC026-SNP31	LOC_Os01g29840	16721109	C/T	INTRON		
ONAC026-SNP32	LOC_Os01g29840	16721129	T/G	INTRON		
ONAC026-SNP33	LOC_Os01g29840	16721348	T/C	URR	NA	-1977
ONAC026-SNP34	LOC_Os01g29840	16721521	G/A	URR	NA	-1804
ONAC026-SNP35	LOC_Os01g29840	16721528	T/A	URR	NA	-1797
ONAC026-SNP36	LOC_Os01g29840	16721588	G/A	URR	NA	-1737
ONAC026-SNP37	LOC_Os01g29840	16721612	G/A	URR	NA	-1713

Supplementary Table S5: Details of SNPs within the selected *NAC* genes. The *cis*- element changes in URR because of an SNP have also been mentioned.						
**SNP IDs**	**Genes**	**Physical position (bp)**	**SNPs**	**Structural annotation**	***Cis*-elements predicted in that region**	**Upstream promoter position**
ONAC026-SNP38	LOC_Os01g29840	16721658	T/C	URR	BIHD1OS, WRKY71OS	-1667
ONAC026-SNP39	LOC_Os01g29840	16721724	C/T	URR	NA	-1601
ONAC026-SNP40	LOC_Os01g29840	16721775	C/T	URR	SITEIIATCYTC	-1550
ONAC026-SNP41	LOC_Os01g29840	16721821	C/T	URR	GATABOX	-1504
ONAC026-SNP42	LOC_Os01g29840	16721844	G/A	URR	NA	-1481
ONAC026-SNP43	LOC_Os01g29840	16721891	G/A	URR	2SSEEDPROTBANAPA, CANBNNAPA	-1434
ONAC026-SNP44	LOC_Os01g29840	16721892	T/C	URR	2SSEEDPROTBANAPA, CANBNNAPA	-1433
ONAC026-SNP45	LOC_Os01g29840	16721928	A/G	URR	CAATBOX1	-1397
ONAC026-SNP46	LOC_Os01g29840	16722082	G/A	URR	_10PEHVPSBD, POLASIG1	-1243
ONAC026-SNP47	LOC_Os01g29840	16722215	C/T	URR	ARR1AT	-1110
ONAC026-SNP48	LOC_Os01g29840	16722236	C/T	URR	NA	-1089
ONAC026-SNP49	LOC_Os01g29840	16722260	A/G	URR	CACTFTPPCA1	-1065
ONAC026-SNP50	LOC_Os01g29840	16722376	A/G	URR	NA	-949
ONAC026-SNP51	LOC_Os01g29840	16722474	C/T	URR	NA	-851
ONAC026-SNP52	LOC_Os01g29840	16722504	T/A	URR	TATABOX3	-821
ONAC026-SNP53	LOC_Os01g29840	16723302	C/T	URR	BIHD1OS	-23
ONAC023-SNP01	LOC_Os02g12310	6402148	T/A	URR	NA	-1934
ONAC023-SNP02	LOC_Os02g12310	6402149	T/C	URR	NA	-1933
ONAC023-SNP03	LOC_Os02g12310	6402153	T/G	URR	NA	-1929
ONAC023-SNP04	LOC_Os02g12310	6402199	A/G	URR	NA	-1883
ONAC023-SNP05	LOC_Os02g12310	6402335	G/A	URR	NA	-1747
ONAC023-SNP06	LOC_Os02g12310	6402395	G/T	URR	NA	-1687
ONAC023-SNP07	LOC_Os02g12310	6402406	G/T	URR	XYLAT, DOFCOREZM	-1676
ONAC023-SNP08	LOC_Os02g12310	6402499	G/A	URR	NA	-1583
ONAC023-SNP09	LOC_Os02g12310	6402522	C/T	URR	TRANSINITDICOTS, TRANSINITMONOCOTS	-1560
ONAC023-SNP10	LOC_Os02g12310	6402549	T/C	URR	POLASIG3, CAATBOX1	-1533
ONAC023-SNP11	LOC_Os02g12310	6402616	A/G	URR	RBCSCONSENSUS, ARR1AT	-1466
ONAC023-SNP12	LOC_Os02g12310	6402805	C/T	URR	DOFCOREZM	-1277

Supplementary Table S5: Details of SNPs within the selected *NAC* genes. The *cis*- element changes in URR because of an SNP have also been mentioned.						
**SNP IDs**	**Genes**	**Physical position (bp)**	**SNPs**	**Structural annotation**	***Cis*-elements predicted in that region**	**Upstream promoter position**
ONAC023-SNP13	LOC_Os02g12310	6402835	C/T	URR	NA	-1247
ONAC023-SNP14	LOC_Os02g12310	6402926	T/A	URR	SEF4MOTIFGM7S, ERELEE4	-1156
ONAC023-SNP15	LOC_Os02g12310	6403097	C/T	URR	NA	-985
ONAC023-SNP16	LOC_Os02g12310	6403112	C/A	URR	GT1CONSENSUS, GT1GMSCAM4	-970
ONAC023-SNP17	LOC_Os02g12310	6403143	A/G	URR	BOXIINTPATPB	-939
ONAC023-SNP18	LOC_Os02g12310	6403154	G/A	URR	CAREOSREP1	-928
ONAC023-SNP19	LOC_Os02g12310	6403159	G/A	URR	NA	-923
ONAC023-SNP20	LOC_Os02g12310	6403211	C/G	URR	GATABOX	-871
ONAC023-SNP21	LOC_Os02g12310	6403222	G/A	URR	NA	-860
ONAC023-SNP22	LOC_Os02g12310	6403226	G/A	URR	NA	-856
ONAC023-SNP23	LOC_Os02g12310	6403305	T/C	URR	TATABOX1	-777
ONAC023-SNP24	LOC_Os02g12310	6403321	C/T	URR	CAATBOX1	-761
ONAC023-SNP25	LOC_Os02g12310	6403338	C/T	URR	CURECORECR	-744
ONAC023-SNP26	LOC_Os02g12310	6403341	G/A	URR	HEXAMERATH4, CGACGOSAMY3, CBFHV, CRTDREHVCBF2	-741
ONAC023-SNP27	LOC_Os02g12310	6403344	G/A	URR	HEXAMERATH4, CGACGOSAMY3, CBFHV, CRTDREHVCBF2	-738
ONAC023-SNP28	LOC_Os02g12310	6403353	A/G	URR	GATABOX	-729
ONAC023-SNP29	LOC_Os02g12310	6403354	C/T	URR	NA	-728
ONAC023-SNP30	LOC_Os02g12310	6403359	G/A	URR	NA	-723
ONAC023-SNP31	LOC_Os02g12310	6403372	C/T	URR	NA	-710
ONAC023-SNP32	LOC_Os02g12310	6403416	G/A	URR	CATATGGMSAUR, EBOXBNNAPA, MYCCONSENSUSAT	-666
ONAC023-SNP33	LOC_Os02g12310	6403449	G/A	URR	NA	-633
ONAC023-SNP34	LOC_Os02g12310	6403459	T/C	URR	NA	-623
ONAC023-SNP35	LOC_Os02g12310	6403469	C/T	URR	EBOXBNNAPA, MYCCONSENSUSAT	-613
ONAC023-SNP36	LOC_Os02g12310	6403477	C/A	URR	CURECORECR, CACTFTPPCA1	-605
ONAC023-SNP37	LOC_Os02g12310	6403485	C/A	URR	BOXIINTPATPB	-597
ONAC023-SNP38	LOC_Os02g12310	6403493	A/T	URR	GT1GMSCAM4	-589

Supplementary Table S5: Details of SNPs within the selected *NAC* genes. The *cis*- element changes in URR because of an SNP have also been mentioned.

SNP IDs	Genes	Physical position (bp)	SNPs	Structural annotation	*Cis* -elements predicted in that region	Upstream promoter position
ONAC023-SNP39	LOC_Os02g12310	6403512	C/G	URR	NA	-570
ONAC023-SNP40	LOC_Os02g12310	6403604	A/C	URR	BOXIINTPATPB, GATABOX	-478
ONAC023-SNP41	LOC_Os02g12310	6403654	G/C	URR	REALPHALGLHCB21, MYB1AT	-428
ONAC023-SNP42	LOC_Os02g12310	6403676	A/T	URR	CAATBOX1	-406
ONAC023-SNP43	LOC_Os02g12310	6403728	T/A	URR	NA	-354
ONAC023-SNP44	LOC_Os02g12310	6403736	C/A	URR	NA	-346
ONAC023-SNP45	LOC_Os02g12310	6403766	C/T	URR	CURECORECR	-316
ONAC023-SNP46	LOC_Os02g12310	6403789	C/A	URR	NA	-293
ONAC023-SNP47	LOC_Os02g12310	6403902	G/A	URR	NA	-180
ONAC023-SNP48	LOC_Os02g12310	6403913	T/A	URR	CAATBOX1	-169
ONAC023-SNP49	LOC_Os02g12310	6404054	A/T	URR	NA	-28
ONAC023-SNP50	LOC_Os02g12310	6404088	A/G	CDS-Synonymous SNP		
ONAC023-SNP51	LOC_Os02g12310	6404103	A/G	CDS-Synonymous SNP		
ONAC023-SNP52	LOC_Os02g12310	6404114	G/A	CDS-Non-Synonymous SNP		
ONAC023-SNP53	LOC_Os02g12310	6404139	G/A	CDS-Synonymous SNP		
ONAC023-SNP54	LOC_Os02g12310	6404157	T/C	CDS-Synonymous SNP		
ONAC023-SNP55	LOC_Os02g12310	6404273	G/A	INTRON		
ONAC023-SNP56	LOC_Os02g12310	6404296	A/G	INTRON		
ONAC023-SNP57	LOC_Os02g12310	6404329	T/C	INTRON		
ONAC023-SNP58	LOC_Os02g12310	6404334	C/T	INTRON		
ONAC023-SNP59	LOC_Os02g12310	6404354	G/A	INTRON		
ONAC023-SNP60	LOC_Os02g12310	6404408	G/A	INTRON		
ONAC023-SNP61	LOC_Os02g12310	6404462	G/A	INTRON		
ONAC023-SNP62	LOC_Os02g12310	6404469	T/C	INTRON		

Supplementary Table S5: Details of SNPs within the selected *NAC* genes. The *cis*- element changes in URR because of an SNP have also been mentioned.						
**SNP IDs**	**Genes**	**Physical position (bp)**	**SNPs**	**Structural annotation**	***Cis*-elements predicted in that region**	**Upstream promoter position**
ONAC023-SNP63	LOC_Os02g12310	6404488	G/A	INTRON		
ONAC023-SNP64	LOC_Os02g12310	6404519	G/A	INTRON		
ONAC023-SNP65	LOC_Os02g12310	6404524	A/G	INTRON		
ONAC023-SNP66	LOC_Os02g12310	6404547	T/C	INTRON		
ONAC023-SNP67	LOC_Os02g12310	6404577	C/T	INTRON		
ONAC023-SNP68	LOC_Os02g12310	6404627	G/A	INTRON		
ONAC023-SNP69	LOC_Os02g12310	6404682	G/A	INTRON		
ONAC023-SNP70	LOC_Os02g12310	6404687	G/A	INTRON		
ONAC023-SNP71	LOC_Os02g12310	6404724	C/T	INTRON		
ONAC023-SNP72	LOC_Os02g12310	6404727	G/A	INTRON		
ONAC023-SNP73	LOC_Os02g12310	6404847	G/T	INTRON		
ONAC023-SNP74	LOC_Os02g12310	6404864	A/G	INTRON		
ONAC023-SNP75	LOC_Os02g12310	6404909	C/T	INTRON		
ONAC023-SNP76	LOC_Os02g12310	6404911	G/A	INTRON		
ONAC023-SNP77	LOC_Os02g12310	6404961	A/G	INTRON		
ONAC023-SNP78	LOC_Os02g12310	6404978	C/T	INTRON		
ONAC023-SNP79	LOC_Os02g12310	6405012	T/C	CDS-Synonymous SNP		
ONAC023-SNP80	LOC_Os02g12310	6405021	G/A	CDS-Synonymous SNP		
ONAC023-SNP81	LOC_Os02g12310	6405044	G/A	CDS-Non-Synonymous SNP		
ONAC023-SNP82	LOC_Os02g12310	6405057	C/T	CDS-Synonymous SNP		
ONAC023-SNP83	LOC_Os02g12310	6405060	A/G	CDS-Synonymous SNP		
ONAC023-SNP84	LOC_Os02g12310	6405094	G/A	CDS-Non-Synonymous SNP		
ONAC023-SNP85	LOC_Os02g12310	6405099	A/C	CDS-Synonymous SNP		

Supplementary Table S5: Details of SNPs within the selected *NAC* genes. The *cis*- element changes in URR because of an SNP have also been mentioned.

SNP IDs	Genes	Physical position (bp)	SNPs	Structural annotation	*Cis* -elements predicted in that region	Upstream promoter position
ONAC023-SNP86	LOC_Os02g12310	6405117	C/T	CDS-Synonymous SNP		
ONAC023-SNP87	LOC_Os02g12310	6405120	C/T	CDS-Synonymous SNP		
ONAC023-SNP88	LOC_Os02g12310	6405186	A/G	CDS-Synonymous SNP		
ONAC023-SNP89	LOC_Os02g12310	6405197	G/A	CDS-Non-Synonymous SNP		
ONAC023-SNP90	LOC_Os02g12310	6405200	A/G	CDS-Non-Synonymous SNP		
ONAC023-SNP91	LOC_Os02g12310	6405201	C/T	CDS-Synonymous SNP		
ONAC023-SNP92	LOC_Os02g12310	6405231	T/C	CDS-Synonymous SNP		
ONAC023-SNP93	LOC_Os02g12310	6405261	G/A	CDS-Synonymous SNP		
ONAC023-SNP94	LOC_Os02g12310	6405272	C/T	CDS-Non-Synonymous SNP		
ONAC023-SNP95	LOC_Os02g12310	6405299	C/T	CDS-Non-Synonymous SNP		
ONAC023-SNP96	LOC_Os02g12310	6405318	G/A	CDS-Synonymous SNP		
ONAC023-SNP97	LOC_Os02g12310	6405357	A/G	CDS-Synonymous SNP		
ONAC023-SNP98	LOC_Os02g12310	6405365	T/A	CDS-Non-Synonymous SNP		
ONAC023-SNP99	LOC_Os02g12310	6405376	G/A	CDS-Non-Synonymous SNP		
ONAC023-SNP100	LOC_Os02g12310	6405476	A/G	CDS-Non-Synonymous SNP		

**Supplementary Table S5: Details of SNPs within the selected *NAC* genes. The *cis-* element changes in URR because of an SNP have also been mentioned.**						
**SNP IDs**	**Genes**	**Physical position (bp)**	**SNPs**	**Structural annotation**	***Cis*-elements predicted in that region**	**Upstream promoter position**
ONAC023-SNP101	LOC_Os02g12310	6405506	C/G	CDS-Non-Synonymous SNP		
ONAC023-SNP102	LOC_Os02g12310	6405508	A/G	CDS-Non-Synonymous SNP		
ONAC023-SNP103	LOC_Os02g12310	6405509	C/A	CDS-Non-Synonymous SNP		
ONAC023-SNP104	LOC_Os02g12310	6405609	T/G	DRR		
ONAC023-SNP105	LOC_Os02g12310	6405648	A/T	DRR		
ONAC023-SNP106	LOC_Os02g12310	6405698	C/A	DRR		
ONAC023-SNP107	LOC_Os02g12310	6405749	T/A	DRR		
ONAC023-SNP108	LOC_Os02g12310	6405770	A/G	DRR		
ONAC023-SNP109	LOC_Os02g12310	6405772	A/G	DRR		
ONAC023-SNP110	LOC_Os02g12310	6405776	T/C	DRR		
ONAC023-SNP111	LOC_Os02g12310	6405790	C/T	DRR		
ONAC023-SNP112	LOC_Os02g12310	6405825	A/G	DRR		
ONAC023-SNP113	LOC_Os02g12310	6405871	T/G	DRR		
ONAC023-SNP114	LOC_Os02g12310	6405907	T/G	DRR		
ONAC023-SNP115	LOC_Os02g12310	6406006	G/A	DRR		
ONAC023-SNP116	LOC_Os02g12310	6406022	A/T	DRR		
ONAC023-SNP117	LOC_Os02g12310	6406031	C/T	DRR		
ONAC023-SNP118	LOC_Os02g12310	6406040	A/G	DRR		
ONAC023-SNP119	LOC_Os02g12310	6406226	A/G	DRR		
ONAC023-SNP120	LOC_Os02g12310	6406346	T/C	DRR		
ONAC023-SNP121	LOC_Os02g12310	6406347	A/G	DRR		
ONAC023-SNP122	LOC_Os02g12310	6406351	T/C	DRR		
ONAC023-SNP123	LOC_Os02g12310	6406395	C/T	DRR		
ONAC023-SNP124	LOC_Os02g12310	6406577	A/G	DRR		
ONAC023-SNP125	LOC_Os02g12310	6406585	G/A	DRR		
ONAC023-SNP126	LOC_Os02g12310	6406599	T/A	DRR		

**Supplementary Table S5: Details of SNPs within the selected *NAC* genes. The *cis*- element changes in URR because of an SNP have also been mentioned.**						
**SNP IDs**	**Genes**	**Physical position (bp)**	**SNPs**	**Structural annotation**	***Cis* -elements predicted in that region**	**Upstream promoter position**
ONAC023-SNP127	LOC_Os02g12310	6406637	G/A	DRR		
ONAC023-SNP128	LOC_Os02g12310	6406642	C/T	DRR		
ONAC023-SNP129	LOC_Os02g12310	6406644	C/T	DRR		
ONAC023-SNP130	LOC_Os02g12310	6406676	G/A	DRR		
ONAC023-SNP131	LOC_Os02g12310	6406677	G/A	DRR		
ONAC023-SNP132	LOC_Os02g12310	6406682	A/G	DRR		
ONAC023-SNP133	LOC_Os02g12310	6406704	A/G	DRR		
ONAC023-SNP134	LOC_Os02g12310	6406717	G/A	DRR		
ONAC023-SNP135	LOC_Os02g12310	6406719	C/T	DRR		
ONAC023-SNP136	LOC_Os02g12310	6406733	G/A	DRR		

Supplementary Table S6: Details of InDels within the selected *NAC* genes. The *cis*-element changes in URR because of an InDel have also been mentioned.

InDel IDs	Genes	Start Physical positions (bp)	End Physical positions (bp)	InDels		Structural annotation	*Cis-* elements predicted in the InDel region	Upstream promoter position
ONAC020-INDEL01	LOC_Os01g01470	239034	239040	ATACTAC	ATAC	URR	CACTFTPPCA1	-1867
ONAC020-INDEL02	LOC_Os01g01470	239037	239039	CTA	CA,CTATA	URR	NA	-1868
ONAC020-INDEL03	LOC_Os01g01470	239253	239263	GAGATAGATAG	GAGATAGATAGATAG,GATAGATAGATAG	URR	NA	-1644
ONAC020-INDEL04	LOC_Os01g01470	239288	239289	TA	TAA	URR	SORLREP3AT, ROOTMOTIFTAPOX1	-1618
ONAC020-INDEL05	LOC_Os01g01470	239319	239321	AAG	A	URR	CACTFTPPCA1	-1586
ONAC020-INDEL06	LOC_Os01g01470	239321	239329	GTATATATA	GTATATATATA	URR	NA	-1578
ONAC020-INDEL07	LOC_Os01g01470	239501	239505	TCCCC	TCCC	URR	NA	-1402
ONAC020-INDEL08	LOC_Os01g01470	239782	239782	C	CATGGCAAAATGTGTAAATTAATTAATATGG	URR	Box 4, TATA-box	-1125
ONAC020-INDEL09	LOC_Os01g01470	239891	239900	GTATATATAT	GTATATATATAT	URR	NA	-1007
ONAC020-INDEL10	LOC_Os01g01470	239954	239957	TACA	TACACA	URR	NA	-950
ONAC020-INDEL11	LOC_Os01g01470	240497	240517	TGAAACAGACCATCGGAAACA	TGAAACA	URR	ANAERO1CONSENSUS, AACACOREOSGLUB1	-390
ONAC020-INDEL12	LOC_Os01g01470	240667	240671	TGGGG	TGGG	URR	NA	-236
ONAC020-INDEL13	LOC_Os01g01470	240703	240710	GTATATAT	GTATATATATAT	URR	NA	-197
ONAC020-INDEL14	LOC_Os01g01470	240831	240843	AGATAGATATATG	AG	URR	NA	-64
ONAC020-INDEL15	LOC_Os01g01470	240851	240861	GGCTATATATG	GG	URR	BIHD10S	-46
ONAC020-INDEL16	LOC_Os01g01470	240887	240892	GAGCAG	GAGCAGCAGCAG	URR	NA	-15

**Supplementary Table S6: Details of InDels within the selected *NAC* genes. The *cis* -element changes in URR because of an InDel have also been mentioned.**								
**InDel IDs**	**Genes**	**Start Physical positions (bp)**	**End Physical positions (bp)**	**InDels**		**Structural annotation**	***Cis-* elements predicted in the InDel region**	**Upstream promoter position**
ONAC020-INDEL17	LOC_Os01g01470	241124	241141	AAATTATATATATAATTA	AAATTA	INTRON		
ONAC020-INDEL18	LOC_Os01g01470	241143	241177	GGAAATTTATATGATATGATATGATGCATGTGAAA	GGAAA	INTRON		
ONAC020-INDEL19	LOC_Os01g01470	241150	241167	TATATGATATGATATGAT	TATATGATATGATATGATATGAT	INTRON		
ONAC020-INDEL20	LOC_Os01g01470	241186	241189	TAAA	TAAAAA	INTRON		
ONAC020-INDEL21	LOC_Os01g01470	241648	241653	CAGATG	C	CDS_FRAME_SHIFT		
ONAC020-INDEL22	LOC_Os01g01470	241722	241728	GCCGCCT	GCCGCCTCCGCCTCCGCCT	CODON_INSERTION		
ONAC020-INDEL23	LOC_Os01g01470	241727	241729	CTT	CT	CDS_FRAME_SHIFT		
ONAC020-INDEL24	LOC_Os01g01470	241728	241728	T	TCCGCC TCCGCCTCCGCCC	CDS_FRAME_SHIFT		
ONAC020-INDEL25	LOC_Os01g01470	241737	241742	CACTCT	C CGACTCT	CDS_FRAME_SHIFT		
ONAC020-INDEL26	LOC_Os01g01470	242066	242084	CTCCATTTTATGCATCCAT	CTCCAT	DRR		
ONAC020-INDEL27	LOC_Os01g01470	242122	242139	GTGTGAGATCGATGTGAG	GTGTGAG	DRR		
ONAC020-INDEL28	LOC_Os01g01470	242154	242154	A	AT	DRR		
ONAC020-INDEL29	LOC_Os01g01470	242155	242168	GATTATTATTATTA	GATTATTATTATTATTA	DRR		
ONAC020-INDEL30	LOC_Os01g01470	242921	242928	GTGACGAA	G	DRR		
ONAC026-INDEL01	LOC_Os01g29840	16718546	16718549	TGGG	TGGGG	DRR		

Supplementary Table S6: Details of InDels within the selected *NAC* genes. The *cis*-element changes in URR because of an InDel have also been mentioned.

InDel IDs	Genes	Start Physical positions (bp)	End Physical positions (bp)	InDels		Structural annotation	*Cis*- elements predicted in the InDel region	Upstream promoter position
ONAC026-INDEL02	LOC_Os01g29840	16718550	16718554	TGGGG	TGGGGG	DRR		
ONAC026-INDEL03	LOC_Os01g29840	16720121	16720126	TAAAAA	TAAAA	DRR		
ONAC026-INDEL04	LOC_Os01g29840	16720185	16720228	CATGTATGTATCTATGTATGTATGTATGTATGTATGTATGTATC	CATGTATGTATGTATGTATGTATC CATGTATGTATGTATGTATGTATGTATC	DRR		
ONAC026-INDEL05	LOC_Os01g29840	16720196	16720227	CTATGTATGTATGTATGTATGTATGTATGTAT	CTATGTATGTATGTATGTATGTAT CTATGTATGTATGTATGTATGTATGTAT CTATGTATGTATGTATGTATGTATGTATGTATGTAT	CDS_FRAME_SHIFT		
ONAC026-INDEL06	LOC_Os01g29840	16720367	16720397	TCTGCTGCTGCTGCTGCTGCTGCTGCTGCTG	TCTGCTGCTGCTGCTGCTG TCTGCTGCTGCTGCTGCTGCTGCTG	CODON_DELETION		
ONAC026-INDEL07	LOC_Os01g29840	16721018	16721023	CCTTCT	CCT	CODON_DELETION		
ONAC026-INDEL08	LOC_Os01g29840	16721049	16721058	ATTTTTTTTT	ATTTTTTTT ATTTTTTTTTT	INTRON		
ONAC026-INDEL09	LOC_Os01g29840	16721090	16721104	TTATATATATATATA	TTATATATATATA TTATATATATATATATA	INTRON		
ONAC026-INDEL10	LOC_Os01g29840	16721096	16721097	AT	A	INTRON		

Supplementary Table S6: Details of InDels within the selected *NAC* genes. The *cis* -element changes in URR because of an InDel have also been mentioned.								
**InDel IDs**	**Genes**	**Start Physical positions (bp)**	**End Physical positions (bp)**	**InDels**		**Structural annotation**	***Cis-* elements predicted in the InDel region**	**Upstream promoter position**
ONAC026-INDEL11	LOC_Os01g29840	16721666	16721670	ACTCT	ACT	URR	NA	-1655
ONAC026-INDEL12	LOC_Os01g29840	16721827	16721830	CTTT	CTT	URR	DOFCOREZM, TAAAGSTKST1	-1495
ONAC026-INDEL13	LOC_Os01g29840	16721932	16721946	TACCATTTGTCTTGA	TA	URR	GT1CONSENSUS, S1FBOXSORPS1L21, EBOXBNNAPA, MYCCONSENSUSAT	-1379
ONAC026-INDEL14	LOC_Os01g29840	16722192	16722197	AGAGGA	AA AGA	URR	NODCON2GM, OSE2ROOTNODULE, PYRIMIDINEBOXHVEPB1	-1128
ONAC026-INDEL15	LOC_Os01g29840	16723295	16723295	T	TC	URR	GATABOX	-30
ONAC023-INDEL01	LOC_Os02g12310	6402120	6402130	TAAAAAAAAAA	TAAAAAAAA,TAAAAAAAAA,TAAAAAAAAAAA	URR	NA	-1952
ONAC023-INDEL02	LOC_Os02g12310	6402292	6402293	TG	T	URR	NA	-1789
ONAC023-INDEL03	LOC_Os02g12310	6402459	6402467	ATTTTTTTT	ATTTTTTT	URR	NA	-1615
ONAC023-INDEL04	LOC_Os02g12310	6402524	6402532	ATTTTTTTT	ATTTTTTT,ATTTTTTTTT,ATTTTTTTTTT	URR	NA	-1550
ONAC023-INDEL05	LOC_Os02g12310	6402532	6402533	TA	TAA,TTAA	URR	NA	-1549
ONAC023-INDEL06	LOC_Os02g12310	6402638	6402641	TCCC	TCC	URR	NA	-1441
ONAC023-INDEL07	LOC_Os02g12310	6403039	6403046	GACGTACG	GACG	URR	CURECORECR	-1036
ONAC023-INDEL08	LOC_Os02g12310	6403843	6403847	TTTTA	T	URR	NA	-235
ONAC023-INDEL09	LOC_Os02g12310	6403857	6403859	AAA	AAACAA	URR	EBOXBNNAPA, MYCCONSENSUSAT	-223

Supplementary Table S6: Details of InDels within the selected *NAC* genes. The *cis* -element changes in URR because of an InDel have also been mentioned.								
**InDel IDs**	**Genes**	**Start Physical positions (bp)**	**End Physical positions (bp)**	**InDels**		**Structural annotation**	***Cis-* elements predicted in the InDel region**	**Upstream promoter position**
ONAC023-INDEL10	LOC_Os02g12310	6403906	6403909	AGGG	AGG	URR	NA	-173
ONAC023-INDEL11	LOC_Os02g12310	6403951	6403952	AG	AGG	URR	NA	-130
ONAC023-INDEL12	LOC_Os02g12310	6403991	6403994	ACCC	ACC	URR	NA	-88
ONAC023-INDEL13	LOC_Os02g12310	6404261	6404270	CTATATATAT	CTATATATATAT	INTRON		
ONAC023-INDEL14	LOC_Os02g12310	6404268	6404272	TATGC	T<br>TAAATGC	INTRON		
ONAC023-INDEL15	LOC_Os02g12310	6404416	6404417	CA	CAA	INTRON		
ONAC023-INDEL16	LOC_Os02g12310	6404418	6404427	GAAAAAAAAA	GAAAAAAAA<br>GAAAAAAAAAA<br>GAAAAAAAAAA<br>A	INTRON		
ONAC023-INDEL17	LOC_Os02g12310	6404552	6404552	A	AG	INTRON		
ONAC023-INDEL18	LOC_Os02g12310	6404607	6404609	ACC	ACCC	INTRON		
ONAC023-INDEL19	LOC_Os02g12310	6404615	6404626	AATACTGATAC<br>T	AATACT	INTRON		
ONAC023-INDEL20	LOC_Os02g12310	6405197	6405212	GCAACAACAA<br>CAACAA	GCAACAACAAC<br>AA	CDS		
ONAC023-INDEL21	LOC_Os02g12310	6405603	6405605	TTT	TTTAATT	DRR		
ONAC023-INDEL22	LOC_Os02g12310	6405648	6405649	AC	A	DRR		
ONAC023-INDEL23	LOC_Os02g12310	6405662	6405667	GAAAAA	GAAAA	DRR		
ONAC023-INDEL24	LOC_Os02g12310	6405775	6405776	GC	G<br>GTC<br>GTTC	DRR		
ONAC023-INDEL25	LOC_Os02g12310	6405776	6405783	CTTTTTTT	CTTTTTTTTT<br>CTTTTTTTTTT	DRR		
ONAC023-INDEL26	LOC_Os02g12310	6405880	6405881	AT	A	DRR		
ONAC023-INDEL27	LOC_Os02g12310	6405925	6405929	ATTTT	ATTT	DRR		
ONAC023-INDEL28	LOC_Os02g12310	6405954	6405957	AATA	AATATA	DRR		
ONAC023-INDEL29	LOC_Os02g12310	6406023	6406023	C	CCG	DRR		
ONAC023-INDEL30	LOC_Os02g12310	6406215	6406217	TGA	T	DRR		

**Supplementary Table S6: Details of InDels within the selected *NAC* genes. The *cis* -element changes in URR because of an InDel have also been mentioned.**								
**InDel IDs**	**Genes**	**Start Physical positions (bp)**	**End Physical positions (bp)**	**InDels**		**Structural annotation**	***Cis-* elements predicted in the InDel region**	**Upstream promoter position**
ONAC023-INDEL31	LOC_Os02g12310	6406439	6406443	TAAAA	TAAA	DRR		

**Supplementary Table S7: Absolute expression levels of the trans-spliced forms of *ONAC020* and *ONAC026***					
**Transcript name**	**S1**	**S2**	**S3**	**S4**	**S5**
ONAC020.C	24.28	26.63	57.66	47.33	29.42
ONAC020.B	27.88	34.60	62.84	48.43	33.74
ONAC026	43.98	51.00	124.46	102.47	80.31
ONAC020.A	43.84	46.86	77.76	60.81	46.93

Supplementary Table S8: Prediction of localization signals of various NAC proteins and their isoforms by four tools as mentioned							
Tool Used	Predicted Organelles	ONAC020				ONAC026	ONAC023
		A	B	C	D		
TargetP	Cp	0.031	0.031	0.031	0.031	0.031	0.078
	Mt	0.414	0.414	0.414	0.414	0.414	0.669
	SP	0.104	0.104	0.104	0.104	0.104	0.124
	AOL	0.868	0.868	0.868	0.868	0.868	0.355
CELLO	PM	0.105	0.206	0.221	0.11	0.23	0.115
	Cy	0.746	0.773	0.66	0.779	0.657	0.941
	Cs	0.051	0.052	0.045	0.025	0.025	0.036
	Ec	0.141	0.151	0.14	0.131	0.136	0.372
	Cp	0.113	0.123	0.107	0.105	0.085	0.413
	Mt	0.311	0.314	0.289	0.243	0.224	0.23
	ER	0.014	0.017	0.014	0.011	0.012	0.058
	Gg	0.017	0.022	0.017	0.014	0.015	0.018
	Ly	0.042	0.041	0.035	0.031	0.026	0.088
	Pr	0.1	0.132	0.099	0.064	0.059	0.217
	Nc	3.341	3.144	3.354	3.469	3.514	2.469
	Vc	0.021	0.024	0.02	0.019	0.018	0.043
WoLF PSORT	Cy						
	Ec						
	Cp		2	1		1	5
	Mt	7	7	7	8	7	4
	Pr						
	Nc	6	4	5	5	5	4
	Vc						
Plant-mPLoc	Nc	√	√	√	√	√	√

The predictions above the cut off values are highlighted in the table. The abbreviations used in the table are listed below.

CW-Cell Wall; PM-Plasma Membrane; Cy-Cytoplasm; Cs-Cytosol; Ec-Extracellular matrix; Cp-Chloroplast; Mt-Mitochondria; ER-Endoplasmic Reticulum; Gg-Golgi Bodies; Ly-Lysosome; Pr-Peroxisome; Pl-Plastids; SP-Secretory pathway; Nc-Nucleus; Vc-Vacuole; AOL-Any Other Location.

References for the tools used:-

1. Nielsen, H., Engelbrecht, J., Brunak, S. & von Heijne, G. Identification of prokaryotic and eukaryotic signal peptides and prediction of their cleavage sites. Protein Eng 10, 1-6 (1997).

2. Emanuelsson, O., Nielsen, H., Brunak, S. & von Heijne, G. Predicting subcellular localization of proteins based on their N-terminal amino acid sequence. J Mol Biol 300, 1005-1016 (2000).

3. Yu, C. S., Lin, C. J. & Hwang, J. K. Predicting subcellular localization of proteins for Gram-negative bacteria by support vector machines based on n-peptide compositions. Protein Sci 13, 1402-1406 (2004).

4. Yu, C. S., Chen, Y. C., Lu, C. H. & Hwang, J. K. Prediction of protein subcellular localization. Proteins 64, 643-651 (2006).

5. Horton, P. et al. WoLF PSORT: protein localization predictor. Nucleic Acids Res 35, W585-W587 (2007).

6. Chou, K. C. & Shen, H. B. Plant-mPLoc: A top-down strategy to augment the power for predicting plant protein subcellular localization. PLoS One 5, e11335 (2010).

7. Chou, K. C. & Shen, H. B. Cell-PLoc: a package of Web servers for predicting subcellular localization of proteins in various organisms. Nat Protoc 3, 153-162 (2008).

8. Chou, K. C. & Shen, H. B. Large-scale plant protein subcellular location prediction. J Cell Biochem 100, 665-678 (2007).

9. Chou, K. C. Using amphiphilic pseudo amino acid composition to predict enzyme subfamily classes. Bioinformatics 21, 10-19 (2005).

10. Shen, H.-B. & Chou, K.-C. Ensemble classifier for protein fold pattern recognition. Bioinformatics 22, 1717-1722 (2006).
