# Supplementary material for: Three Rice NAC Transcription Factors Heteromerize and Are Associated with Seed Size
Source: Front Plant Sci. 2016 Nov 7;7:1638. doi: 10.3389/fpls.2016.01638 (PMC5098391; doi:10.3389/fpls.2016.01638)
Supplement: Supplementary file 2 [file Image_1.PDF]

Title

THREE RICE NAC TRANSCRIPTION FACTORS HETEROMERIZE AND ARE ASSOCIATED WITH SEED SIZE

Running Title

Role-play amongst NAC family members

Authors

Iny Elizebeth Mathew, Sweta Das, Arunima Mahto, Pinky Agarwal*

Affiliation

National Institute of Plant Genome Research, Aruna Asaf Ali Marg, New Delhi-110067, India

Correspondence

Pinky Agarwal

(pinky.agarwal@nipgr.ac.in)

Supplementary Figure S1. Expression analysis of NAC genes. Heat map of seed-specific *NAC* genes from Rice Oligonucleotide Array Database (ROAD; http://www.ricearray.org/) in the development stages, as mentioned on the top. The selected genes have been marked in red and the colour legend shows the expression levels, with blue, black and yellow indicating no, medium and high expression levels, respectively.

Supplementary Figure S2. A phylogenetic tree of all NAC TFs from rice (Nuruzzaman *et al*., 2010) with the three selected genes marked in blue boxes.

Supplementary Figure S3. Mean seed weight for five rice accessions. The accessions considered are SN, PB1, IR64, NB and LGR and are represented on the X-axis. The average of 1000 seed weight, for each is plotted on the Y-axis. The bars representing standard deviation of three independent replicates have been marked.

Supplementary Figure S4. Distribution of *NAC* gene-derived sequence variants on the rice chromosome as depicted by the Circos circular ideogram. The circle represents twelve rice chromosomes as color coded differently, with the numbers denoting the physical size in Mb. The non-coding, non-synonymous and synonymous SNPs are in black and yellow and green colored dots respectively. All the InDels, both in the coding and non-coding region are represented by red colored dots.

Supplementary Figure S5. (A) Normal distribution of the grain weight trait variation among the 192 rice accessions. The mean value of the grain weight was found to be 26.5g ± 4.8 with 18.1% coefficient of variation and a H^2 value of 80%. (B) The unbiasedness of the mapping population is depicted by a Q-Q plot.

Supplementary Figure S6. Validation of the sequence variants associated with the grain weight trait in the mapping population depicting InDel 01 in (A) *ONAC020*, (B) SNP50 in *ONAC026* and (C) SNP15 in *ONAC023*. (D) Confirmation that the sequence variants identified in *ONAC026* covered the entire gene without LD decay.

Supplementary Figure S7. Transcript fusion or trans-splicing between *ONAC020* and *ONAC026*. A) Alignment of *ONAC020.A* and *ONAC026* with the transcript fusions (B, C and D) showing the primers used for amplification of three forms A, B and C (marked in red dotted box, gel photograph in C). B) Line diagram showing the similar regions between *ONAC020* and *ONAC026* and the regions of fusion between the two amongst the various transcripts, as indicated in the legend. *ONAC020* has two introns and the six transcripts (A to F) have different regions of insertion or deletion, which are due to transcript fusion with *ONAC026*. C) Gel photograph shows the relative levels of trans-spliced forms of ONAC020 and ONAC026 in five different stages of seed development designated S1-S5. The semi-quantitative RT-PCR was performed with a common primer set, as marked in (A) which shows clear bands for forms A (217 bp, red dot), B (241 bp, yellow dot) and C (253 bp, blue dot). *ONAC026* (green dot) is also amplified because of high homology with *ONAC020*. M is O'GeneRuler™ 50 bp DNA ladder from Fermentas® and N is the negative control.

Supplementary Figure S8. Transcriptional activation/repression and growth assay of NAC TFs. A and C) transactivation and B and D) transrepression assays of the three NAC proteins, and the proteins from transcript fusion of *ONAC020/ONAC026* (ie. ONAC020.B to D), by levels of activation of l*acZ* gene (A and B) on SD media lacking tryptophan, with 80 mg/L of X-α-gal as substrate. The intensity of blue color relates with the level of activation/repression with repect to the control. C) and D) shows the activation of *ADE2* and *HIS3* reporter genes on SD media lacking tryptophan, adenine and histidine supplemented with 10 mM 3-AT. The growth of the yeast cells relate with the level of activation. The vector pGBKT7 is the negative control for transactivation assays in (A and C) and the reconstituted GAL4 TF (rGAL4) is the positive control for transrepression assays in (B and D). E) The ability of the proteins to effect the growth of yeast cells was tested by plating various dilutions, as mentioned above the figure. The starting $O.D._{600}$ was fixed at 0.2 and the cells were grown on SD media lacking tryptophan. The protein names are mentioned on the right side and the vector control was pGBKT7.

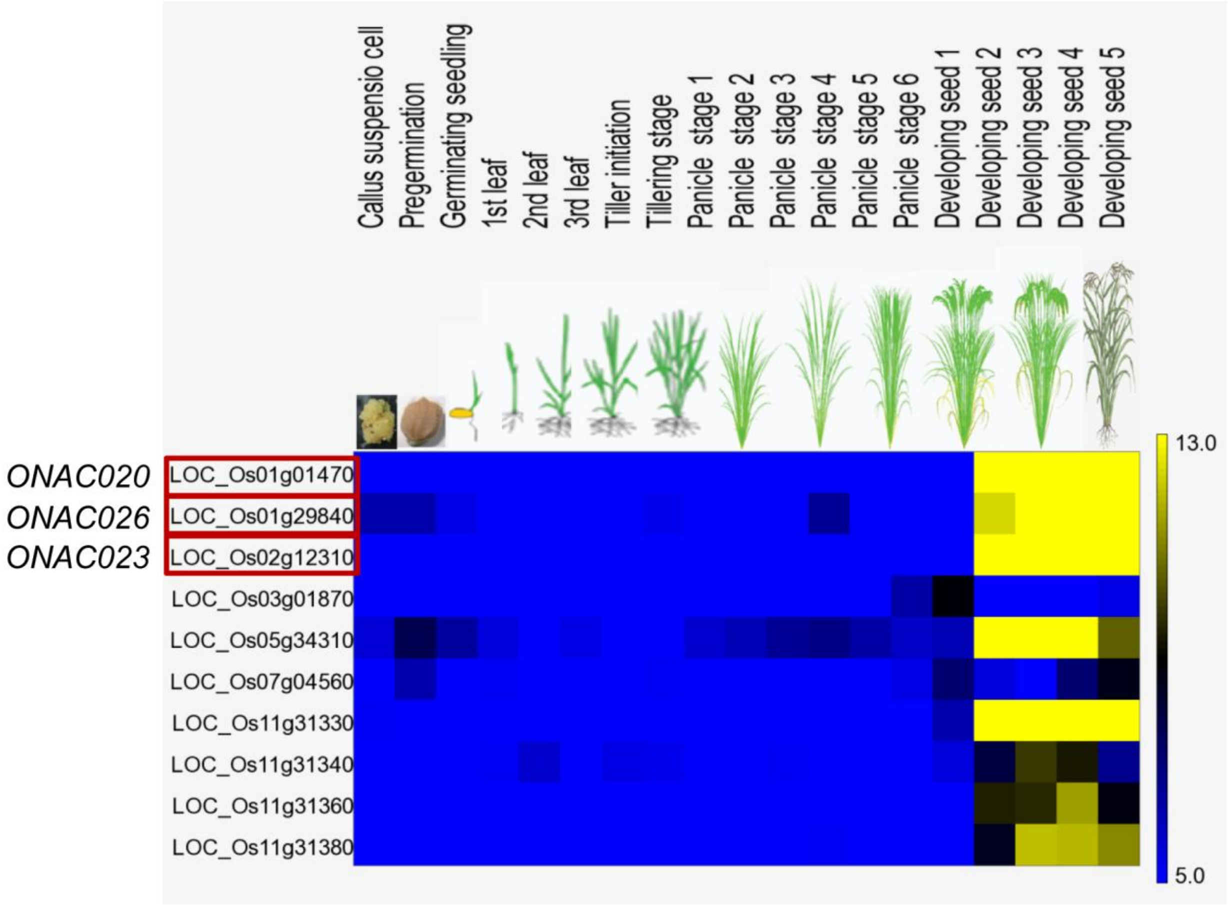

Supplementary Figure S1

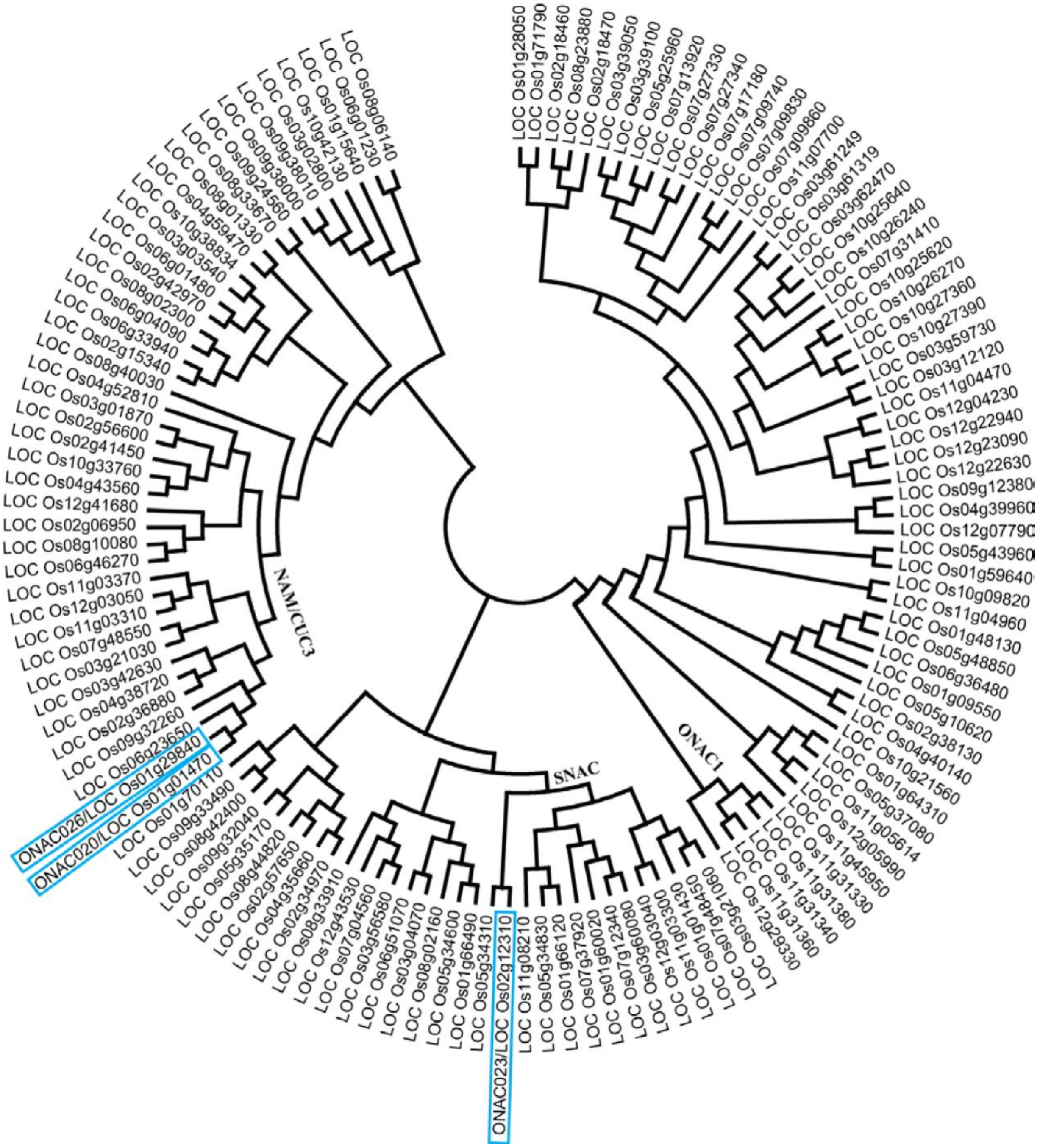

Supplementary Figure S2

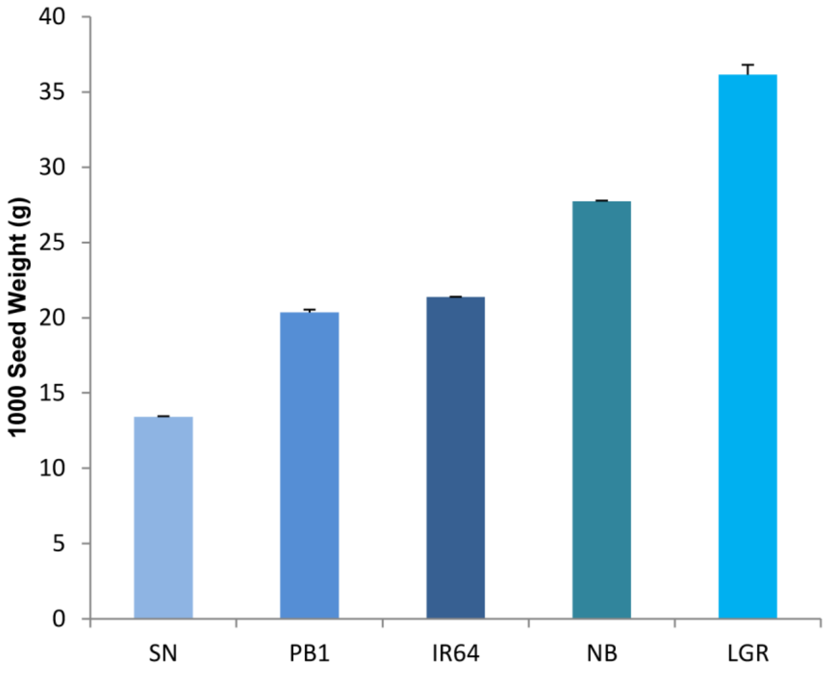

Supplementary Figure S3

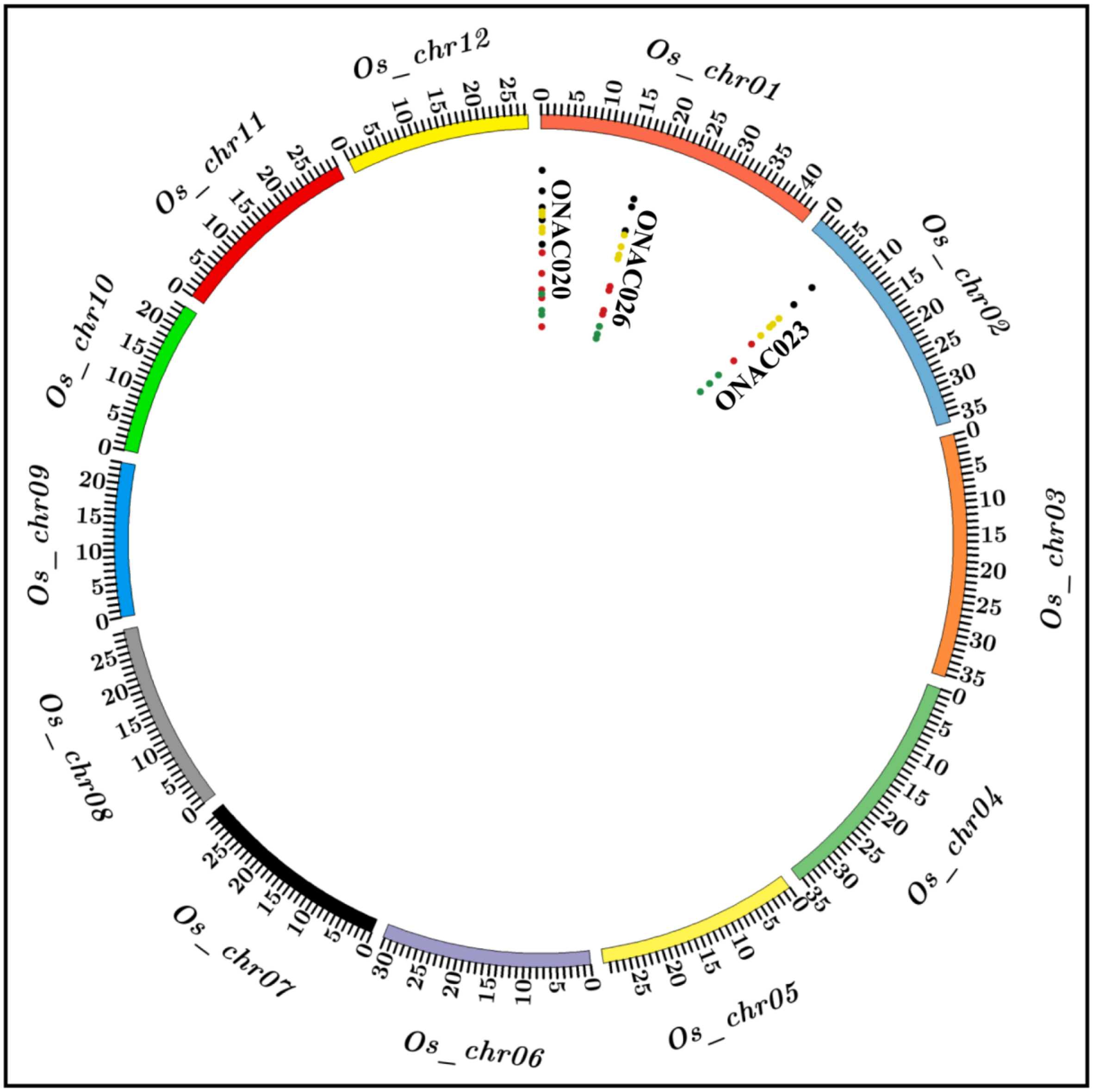

Supplementary Figure S4

(A)

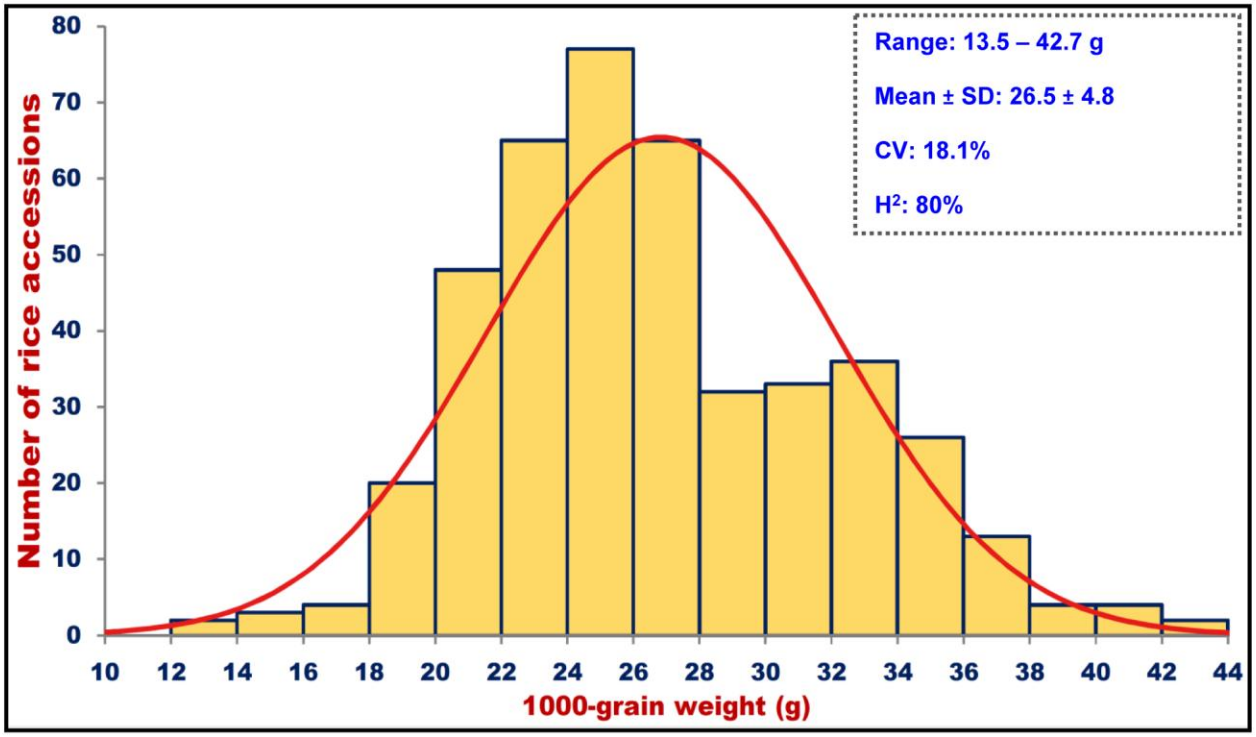

(B)

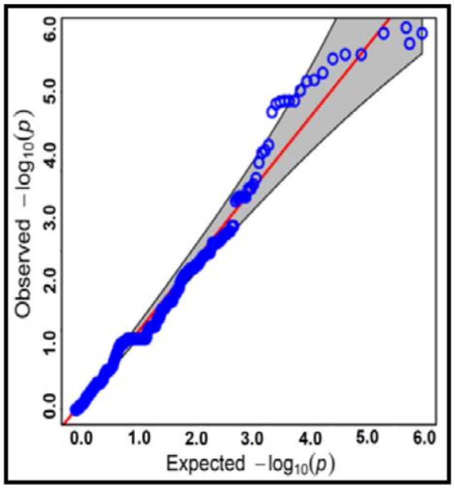

Supplementary Figure S5

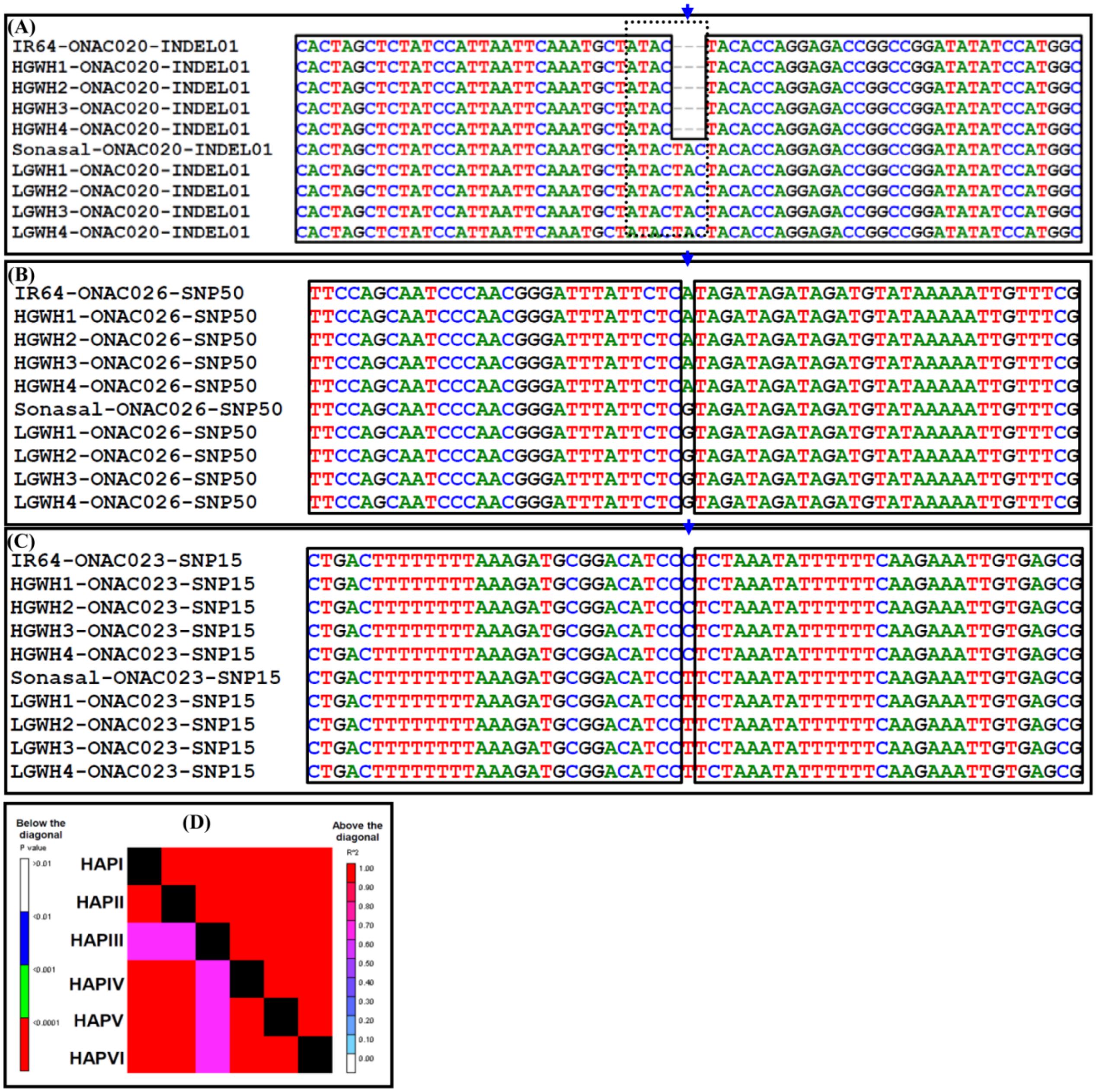

Supplementary Figure S6

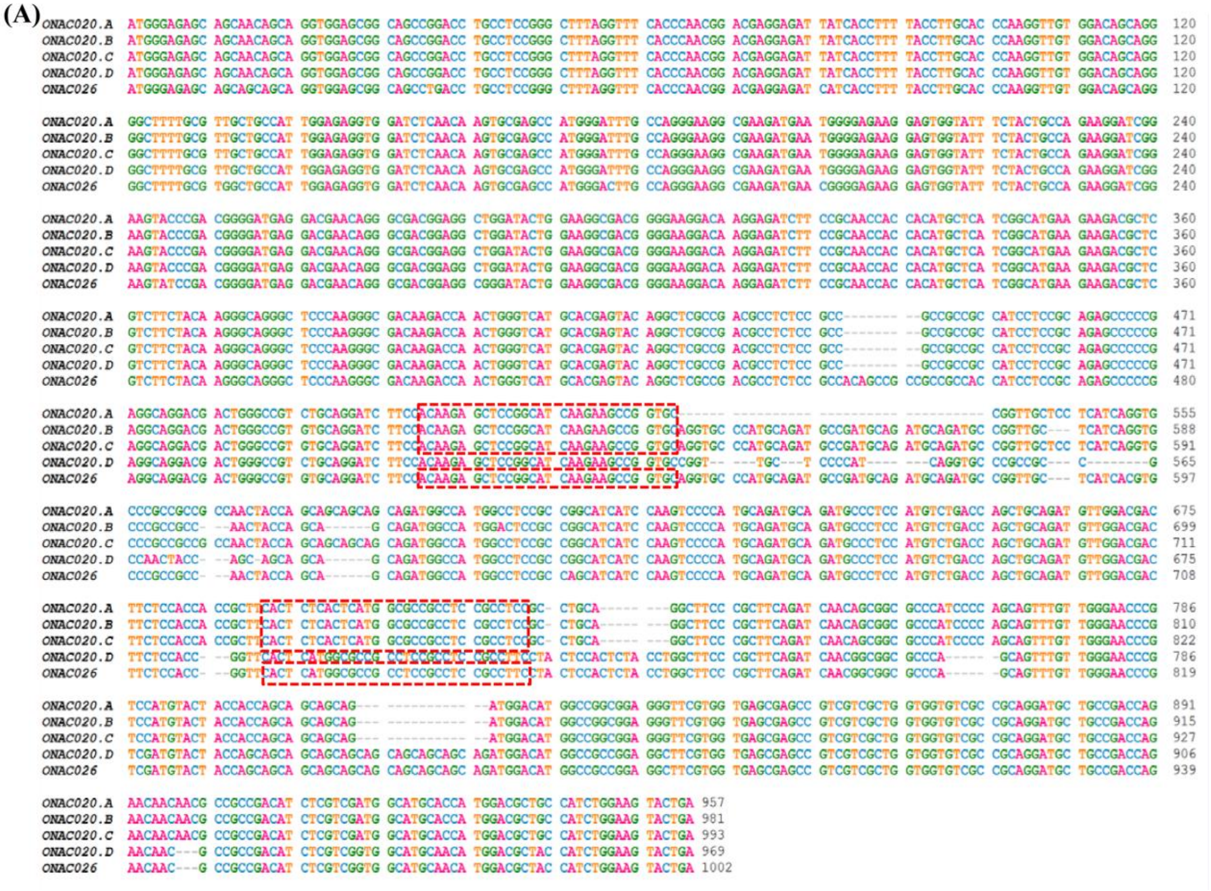

Supplementary Figure S7 (continued…)

(B)

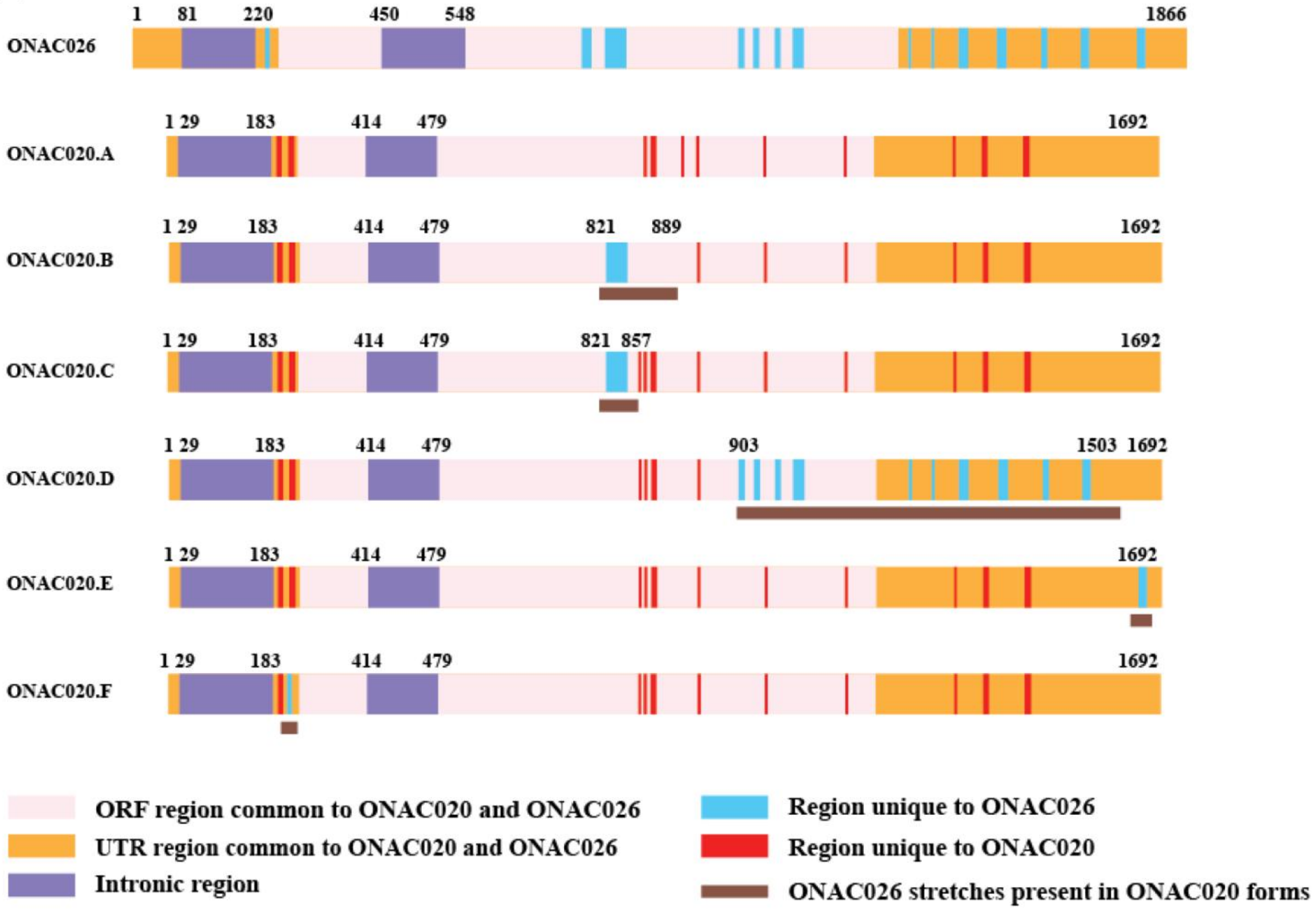

(C)

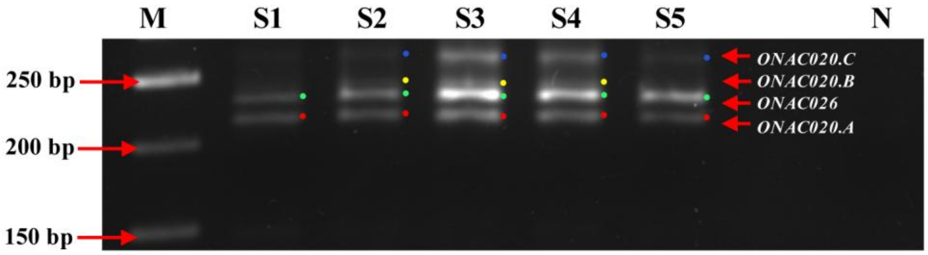

(…continued) Supplementary Figure S7

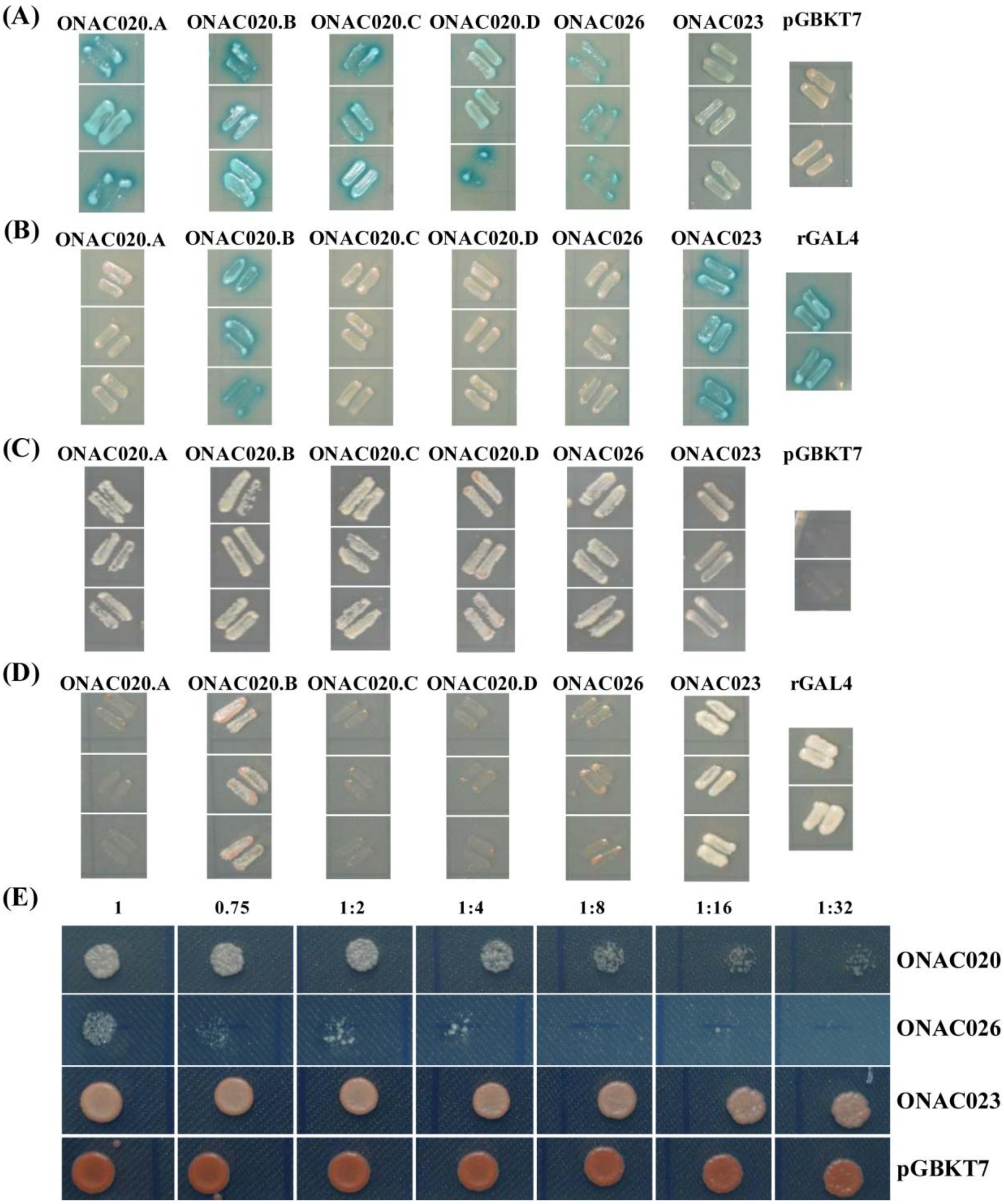

Supplementary Figure S8
